# Acid-Responsive Zeolitic Imidazolate Framework-8 Core–Shell Nanovesicles Loaded with Interleukin-2 Nanoplatforms Selectively Expand Regulatory T Cells to Reconstruct Immunometabolic Homeostasis in Diabetic Neuropathy

**DOI:** 10.34133/bmr.0407

**Published:** 2026-09-04

**Authors:** Yangjie Li, Yanqing Liu, Fei Gong, Kangling Xie, Ling Zhang, Taolin Fan, Cui Li, Jiahao Li, Fan Hu, Ying Cai

**Affiliations:** ^1^ Department of Rehabilitation, National Clinical Research Center for Geriatric Disorders, Xiangya Hospital Central South University, Changsha 410008, Hunan Province, China.; ^2^ Hunan Xiangya Boai Rehabilitation Hospital Co., Ltd., Changsha 411000, Hunan Province, China.; ^3^ Department of Rehabilitation Medicine, Xiangya Hospital, Central South University (National Regional Center for Neurological Diseases), Nanchang 330038, Jiangxi Province, China.

## Abstract

This study presents a nanotherapeutic strategy for diabetic peripheral neuropathy (DPN) by developing polyimide-coated zeolitic imidazolate framework-8 core–shell nanovesicles loaded with interleukin-2 (MOF@PI-IL2) for inflammation-microenvironment-responsive delivery of IL-2 and relatively preferential activation of regulatory T cells (Treg cells). Given that DPN is characterized by chronic neuroinflammation and immune dysregulation with impaired Treg cell numbers and activity, the engineered nanoparticles promote Treg cell expansion and immunometabolic reprogramming while suppressing CD8^+^ T cell cytotoxicity. Through comprehensive evaluation using multiomics integration, biomineralization synthesis, inflammatory models, and DPN mouse experiments, the system demonstrated favorable physicochemical properties and biodistribution. Treatment with MOF@PI-IL2 significantly improved both electrophysiological parameters and structural nerve function. Forkhead box P3 knockdown experiments confirmed that the therapeutic effects were Treg cell dependent. Multiomics analysis further suggested that the treatment partly corrected short-chain fatty acid and tryptophan metabolic dysregulation and was associated with reconstruction of correlations within a “metabolism–IL-2 response–Treg cell module”. This work provides an inflammation-microenvironment-responsive and translatable Treg-cell-targeted nanoimmunotherapeutic strategy for DPN while broadening the conceptual framework for treating chronic inflammatory diseases through immunometabolic modulation.

## Introduction

Diabetic peripheral neuropathy (DPN) is one of the most common chronic complications of diabetes, with high incidence and disability rates that seriously impair patients’ quality of life [1]. Clinically, DPN presents with complex manifestations, including sensory loss, pain, motor dysfunction, and autonomic impairment, reflecting its multifactorial pathogenesis [2]. Beyond metabolic abnormalities, growing evidence suggests that chronic low-grade neuroinflammation and immune dysregulation are key drivers of DPN initiation and progression [3,4]. Current treatments mainly focus on glycemic control, neurotrophic support, and pain relief [5], yet effective therapies targeting the underlying pathogenic mechanisms remain limited. Consequently, DPN management is still largely restricted to symptomatic relief rather than disease-modifying intervention, highlighting the urgent need for new therapeutic strategies based on immunopathological mechanisms [6].

Among immunoregulatory mechanisms, regulatory T cells (Treg cells), which are essential for maintaining immune homeostasis, have attracted increasing attention for their roles in neurodegenerative and chronic inflammatory diseases [7–9]. Treg cells exert immunosuppressive functions through expression of the transcription factor forkhead box P3 (Foxp3), inhibition of overactivated effector T cells, and secretion of anti-inflammatory cytokines such as interleukin-10 (IL-10) and transforming growth factor-β (TGF-β), thereby attenuating local inflammatory responses [10,11]. In addition, Treg cells contribute to neuroprotection in both the central and peripheral nervous systems by modulating glial activation and promoting nerve repair [12]. However, under diabetic conditions, persistent hyperglycemia, oxidative stress, and a proinflammatory microenvironment can reduce Treg cell abundance and impair their function, thereby aggravating neuroinflammation and tissue injury [13,14]. Restoring Treg cell function has been shown to suppress proinflammatory mediator release, alleviate neuroinflammation, and improve nerve conduction, underscoring the therapeutic potential of Treg-cell-targeted approaches in DPN [15].

In recent years, IL-2 has emerged as a promising immunotherapeutic agent because of its central role in sustaining Treg cell proliferation and function [16,17]. Low-dose IL-2 can selectively expand Treg cells without markedly activating other immune cell populations, offering favorable specificity and safety profiles [18]. However, its poor in vivo stability, short half-life, and risk of nonspecific immune activation following systemic administration greatly limit its clinical application [19]. These challenges highlight the need for a safe, efficient, and targeted IL-2 delivery platform. Metal–organic frameworks (MOFs), an emerging class of nanomaterials, have been widely explored for targeted drug delivery because of their high surface area, tunable structure, strong drug-loading capacity, and pH-responsive properties [20,21]. Polymer coating or surface functionalization can further improve MOF stability and confer stimulus-responsive behavior, enabling more precise drug release [22–24]. Under diabetes and other chronic inflammatory conditions, lesions often exhibit a mildly acidic microenvironment, which may serve as an endogenous trigger for responsive drug delivery [25,26]. Similarly, the mildly acidic inflammatory milieu in DPN may provide a suitable trigger for localized release. Therefore, polymer-modified MOF nanostructures represent a promising strategy for site-specific immunomodulatory delivery.

Based on this background, the present study aimed to develop a polyimide (PI)-coated zeolitic imidazolate framework-8 core–shell nanovesicles loaded with IL-2 (MOF@PI-IL2) nanovesicle system with controlled-release properties, focusing on inflammation-microenvironment-responsive IL-2 delivery and restoration of Treg cell function, thereby systematically modulating the dysregulated immune microenvironment and neuropathological state in DPN.

## Materials and Methods

### Biomineralization-based fabrication of IL-2-loaded zeolitic imidazolate framework-8 nanoparticle core

Recombinant human IL-2 (rhIL-2; PeproTech, 200-02; preservative-free) was dissolved in sterile deionized water to 50 μg/ml. Subsequently, 2-methylimidazole (Sigma-Aldrich, M50834) and zinc nitrate hexahydrate [Zn(NO_3_)_2_·6H_2_O; Sigma-Aldrich, 228737] were sequentially added at a molar ratio of 1:8. The reaction mixture was maintained at pH 7.5 and shaken at 25 °C for 30 min to enable synchronous crystallization and encapsulation of IL-2, yielding IL-2-loaded zeolitic imidazolate framework-8 (IL-2@ZIF-8) nanoparticles. The nanocrystals were collected by centrifugation at 12,000*g* for 10 min, washed 3 times with sterile phosphate-buffered saline (PBS; Gibco, 10010023), and resuspended for further use.

### Interfacial condensation and ultrathin coating of PI shell

Pyromellitic dianhydride (Sigma-Aldrich, 368229) and 4,4-diaminodiphenyl ether (Sigma-Aldrich, A65206), used as PI precursors, were each prepared at 10 mg/ml and sterilized through 0.22-μm filters. The IL-2@ZIF-8 suspension was first dispersed in the 4,4-diaminodiphenyl ether solution, and pyromellitic dianhydride was then added dropwise under magnetic stirring (600 rpm) for 40 min. After interfacial cross-linking, the product was washed 3 times by centrifugation at 10,000*g* to obtain MOF@PI-IL2 nanovesicles with a continuous ultrathin PI shell.

### Transmission electron microscopy characterization

For transmission electron microscopy (TEM), nanovesicles were dropped onto carbon-coated copper grids, air-dried, and imaged using a Tecnai G2 Spirit TEM (FEI) at 120 kV. Particle morphology and structural integrity were analyzed using ImageJ (National Institutes of Health).

### Scanning electron microscopy characterization

For scanning electron microscopy (SEM), MOF@PI-IL2 suspensions were diluted to 0.1 mg/ml, deposited onto silicon wafers, air-dried, sputter-coated with gold, and observed using an Apreo S field-emission SEM (Thermo Fisher Scientific) at 3 kV. Surface morphology was analyzed under identical acquisition settings [27].

### Dynamic light scattering and zeta potential measurement

Hydrodynamic diameter, polydispersity index (PDI), and zeta potential were measured using a Zetasizer Nano ZS90 (Malvern Panalytical). Samples were diluted in PBS to 0.2 mg/ml and analyzed at 25 °C. Each sample was measured in triplicate.

### Quantification of IL-2 loading and encapsulation efficiency

Unencapsulated IL-2 was quantified using a human IL-2 enzyme-linked immunosorbent assay (ELISA) kit (R&D Systems, DY202). Filtrates were collected with Amicon Ultra-10K centrifugal filters (Millipore), and absorbance was measured at 450 nm using a SpectraMax iD3 microplate reader (Molecular Devices). Drug loading and encapsulation efficiency were calculated by subtracting the unbound IL-2 from the initial input.

### Ultraviolet–visible absorption spectrum

Ultraviolet–visible (UV–Vis) spectra of IL-2, IL-2@ZIF-8, and MOF@PI-IL2 were acquired using a UV-2600i spectrophotometer (Shimadzu). All samples were measured in quartz cuvettes (Hellma, QS-1) over a wavelength range of 200 to 400 nm with a step size of 1 nm and an integration time of 0.5 s. The retention of IL-2 characteristic peaks indicated the integrity of the encapsulation.

### Attenuated total reflectance–Fourier transform infrared spectral analysis

Characteristic peaks of the PI shell and ZIF-8 framework were obtained using a Nicolet iS50 Fourier transform infrared (FTIR) spectrometer (Thermo Fisher Scientific) equipped with a diamond attenuated total reflectance crystal platform. Each sample was scanned 32 times at room temperature, with a spectral resolution of 4 cm^−1^ over the range 400 to 4,000 cm^−1^. Symmetric and asymmetric stretching vibrations of the imide group, along with Zn–N stretching bands, were used to confirm successful integration of the 3-component structure.

### Colloidal stability and particle size distribution of MOF@PI-IL2 under varying pH conditions

To evaluate colloidal stability, MOF@PI-IL2 suspensions (0.2 mg/ml) were incubated in PBS at 37 °C for up to 72 h. Samples were collected at 0, 6, 12, 24, 48, and 72 h, and particle size and PDI were measured by dynamic light scattering (DLS; Zetasizer Nano ZS90, Malvern Panalytical, ZEN3690). Stability was assessed according to the degree of size fluctuation and maintenance of low PDI values.

### X-ray diffraction characterization of MOF@PI-IL2 crystalline structure after pH treatments

MOF@PI-IL2 suspensions (1 mg/ml) were incubated in PBS (pH 7.4) or Hepes buffer (pH 6.5; Solarbio, H1042) at 37 °C for 48 h. After incubation, samples were centrifuged at 12,000*g* to collect the precipitates, which were washed 3 times with deionized water and dried overnight in a vacuum oven at 40 °C. The resulting powders were analyzed using an x-ray diffractometer (D8 Advance, Bruker) with Cu Kα radiation (*λ* = 1.5406 Å) operated at 40 kV and 40 mA. Diffraction patterns were recorded over a 2*θ* range of 5 to 40° with a step size of 0.02° and an integration time of 0.5 s. The characteristic diffraction peaks of ZIF-8 were compared between the 2 pH conditions.

### Time-resolved TEM imaging and quantification of PI shell thickness

MOF@PI-IL2 suspensions (0.5 mg/ml) were incubated in PBS (pH 7.4) or Hepes buffer (pH 6.5) at 37 °C, and samples were collected at 0, 24, and 48 h. At each time point, particles were collected by centrifugation at 10,000*g*, resuspended in deionized water, deposited onto carbon-coated copper grids (Electron Microscopy Sciences, CF200-Cu), and air-dried. TEM imaging was performed using a Tecnai G2 Spirit microscope (FEI) at 120 kV under identical magnification and exposure settings. Shell continuity and morphology were evaluated from high-magnification images, and shell thickness was measured in ImageJ (National Institutes of Health). For each condition, 50 randomly selected particles were analyzed from multiple orientations, and the average shell thickness was calculated.

### pH-dependent IL-2 release kinetics

The in vitro release profile of IL-2 from MOF@PI-IL2 was evaluated using a dialysis method. MOF@PI-IL2 suspensions containing 5 μg of IL-2 equivalent were loaded into dialysis bags with a 10-kDa molecular weight cutoff (Spectrum Labs, 132130) and immersed in 10 ml of PBS (pH 7.4) or Hepes buffer (pH 6.5). The samples were incubated at 37 °C with shaking at 80 rpm. At 0, 2, 4, 8, 12, 24, 36, and 48 h, 200 μl of the external medium was collected and replaced with an equal volume of fresh buffer. Released IL-2 was quantified using a human IL-2 ELISA kit (R&D Systems, DY202), and absorbance was measured at 450 nm with a SpectraMax iD3 microplate reader (Molecular Devices). IL-2 concentrations were calculated from a standard curve, and cumulative release was expressed as a percentage of the initial loading. Release curves were plotted using GraphPad Prism 10.

### Cell sorting and culture

Mouse spleens were dissociated through 70-μm cell strainers (Corning, 352350), and CD4^+^CD25^+^ cells were preenriched using the Mouse CD4^+^CD25^+^ Treg Cell Isolation Kit (Miltenyi Biotec, 130-091-041). High-purity CD4^+^CD25^+^Foxp3^+^ Treg cells and CD4^+^CD25^−^ non-Treg cells were further sorted using a FACSAria III cell sorter (BD Biosciences). Cells were cultured in RPMI 1640 medium (Gibco, 11875093) supplemented with 10% fetal bovine serum (Gibco, 10099141C) and 1% penicillin–streptomycin (Gibco, 15140122), and all experiments were performed within 24 h after sorting.

### Preparation of DiI-labeled MOF@PI-IL2

Preformed MOF@PI-IL2 nanovesicles were labeled with Vybrant DiI Cell-Labeling Solution (Thermo Fisher Scientific, V22885) in the dark at 37 °C for 20 min. Unbound dye was removed by 3 rounds of centrifugal washing at 12,000*g* using Amicon Ultra-10K filters (Millipore, UFC501024). The integrity of the labeled nanovesicles was verified by absorbance spectroscopy using a NanoDrop OneC (Thermo Fisher Scientific).

### Treg-cell-selective uptake observed by confocal imaging

Treg and non-Treg cells were seeded at a density of 1 × 10^5^ cells per well in 35-mm glass-bottom dishes (Cellvis, D35-10-1.5-N) and incubated with DiI (1,1′-dioctadecyl-3,3,3′,3′-tetramethylindocarbocyanine perchlorate)-labeled MOF@PI-IL2 (DiI-MOF@PI-IL2) (100 μg/ml) for 4 h at 37 °C in 5% CO_2_. Cells were then fixed with 4% paraformaldehyde (Biosharp, BL539A) for 10 min and counterstained with 4′,6-diamidino-2-phenylindole (DAPI; Abcam, ab228549). Confocal images were acquired using a Leica TCS SP8 microscope (Leica Microsystems). All imaging parameters were kept constant across groups.

### Quantification of fluorescence intensity at the single-cell level

For each group, at least 50 cells were randomly selected for analysis. Cytoplasmic regions were manually outlined in Fiji (ImageJ 1.53c), and mean fluorescence intensity was measured after background subtraction. Fluorescence values were normalized to those of the non-Treg-cell group.

### Time-lapse imaging of endocytic kinetics

Cells were incubated with DiI-MOF@PI-IL2 for 0.5, 1, 2, and 4 h. Confocal images were acquired at each time point using identical laser power, gain, and scan speed settings. Fluorescence intensity over time was fitted to a one-phase association model in GraphPad Prism 10 to estimate the half-time of internalization (*t*_1_/_2_).

### Colocalization analysis of endocytic pathways

Cells were incubated with primary antibodies against early endosome antigen-1 (EEA1; Abcam, ab109110; 1:200) or Ras-related protein Rab-7a (Rab7) (Abcam, ab137029; 1:200) at 37 °C for 1 h. For lysosomal labeling, LysoTracker Green DND-26 (Thermo Fisher Scientific, L7526; 50 nM) was applied for 30 min. Cells were then coincubated with DiI-MOF@PI-IL2 (100 μg/ml) for 1 to 4 h. After incubation, cells were fixed with 4% paraformaldehyde for 15 min, permeabilized with 0.2% Triton X-100 for 10 min, and blocked with 5% bovine serum albumin for 1 h. Alexa-Fluor-488-conjugated goat anti-rabbit immunoglobulin G (Thermo Fisher Scientific, A-11008; 1:500) was used as the secondary antibody, and nuclei were counterstained with DAPI (Sigma-Aldrich, D9542). Images were acquired using a Leica SP8 confocal microscope (Leica Microsystems) under identical imaging settings. Colocalization was analyzed using the Coloc2 plugin in Fiji (ImageJ), and Pearson’s correlation coefficient was calculated for each group.

### Treg cell cytotoxicity assessment

Treg cells were seeded in 96-well plates at a density of 1 × 10^4^ cells per well and treated with MOF@PI-IL2 at concentrations ranging from 0 to 200 μg/ml for 24 h. Cell viability was then assessed using the Cell Counting Kit-8 (CCK-8; Dojindo, CK04). Briefly, 10 μl of CCK-8 reagent was added to each well, followed by incubation at 37 °C for 2 h. Absorbance was measured at 450 nm using a SpectraMax iD3 microplate reader (Molecular Devices). Cell viability was normalized to that of the untreated control group.

### Establishment and grouping of a lipopolysaccharide-induced Treg cell suppression model

Primary mouse CD4^+^ T cells were isolated via negative selection using the Miltenyi CD4^+^ T Cell Isolation Kit (Miltenyi Biotec, 130-104-454) and subsequently activated with Dynabeads Mouse T-Activator CD3/CD28 (Thermo Fisher Scientific, 11456D) at a 1:1 ratio for 24 h. The cells were then stimulated with lipopolysaccharide (LPS; Sigma-Aldrich, L2630; 100 ng/ml) for an additional 24 h to establish an inflammatory suppression model. Five experimental groups were defined: control, LPS, LPS + MOF@PI, LPS + free IL-2, and LPS + MOF@PI-IL2. Both MOF@PI and MOF@PI-IL2 were administered at a final concentration of 50 μg/ml, while free IL-2 (rhIL-2; PeproTech, 200-02) was used at 10 ng/ml. All experiments were conducted in a 37 °C, 5% CO_2_ incubator (Thermo Fisher Scientific, Heracell 150i).

### siRNA-mediated Foxp3 knockdown in Treg cells

Foxp3 knockdown (Foxp3 KD) was performed in mouse-derived Treg cells cultured in antibiotic-free RPMI 1640 medium (Gibco, 11875093). A pool of 3 siRNA duplexes targeting mouse Foxp3 was used: GACAGAAGAAAGCTTAGAGAAGA, TCCACAACATGGACTACTTCAAG, and TCGTGAACTTTCAAAGTTAGTCA. Transfection was carried out using Lipofectamine RNAiMAX (Invitrogen, 13778030). Briefly, 30 pmol of siRNA was mixed with 1.5 μl RNAiMAX in Opti-MEM (Gibco, 31985070), incubated at room temperature for 10 min, and then added to the cells. After 48 h of incubation at 37 °C in 5% CO_2_, cells were harvested for protein extraction and subsequent functional assays.

### Flow cytometry analysis of Treg cell (CD4^+^CD25^+^Foxp3^+^) proportions

Cells were fixed and permeabilized using the Fixation/Permeabilization Buffer Set (eBioscience, 00-5523-00), followed by staining with anti-CD4–fluorescein isothiocyanate (BioLegend, 100510), anti-CD25–allophycocyanin (APC) (BioLegend, 102012), and anti-Foxp3–phycoerythrin (eBioscience, 12-5773-82) at 1:200 dilution for 30 min at 4 °C in the dark. Samples were acquired on a BD LSRFortessa flow cytometer (BD Biosciences), with 50,000 events collected per sample. Data were analyzed in FlowJo v10 using a standard gating strategy, and Treg cells were defined as CD4^+^CD25^+^Foxp3^+^ cells.

### Western blot analysis

Cells or sciatic nerve tissues were lysed in radioimmunoprecipitation assay buffer (Beyotime, P0013B) containing protease and phosphatase inhibitors (Roche, 04693159001). Protein concentrations were determined using a bicinchoninic acid assay kit (Thermo Fisher Scientific, 23227). Equal amounts of protein (20 μg) were separated by 10% or 12% SDS-polyacrylamide gel electrophoresis and transferred onto polyvinylidene difluoride membranes (Millipore, IPVH00010). Membranes were blocked with 5% bovine serum albumin for 1 h at room temperature and incubated overnight at 4 °C with primary antibodies against phosphorylated signal transducer and activator of transcription 5 (p-STAT5; Cell Signaling Technology, 9351; 1:1,000), STAT5 (Cell Signaling Technology, 94205; 1:1,000), Foxp3 (Abcam, ab215206; 1:1,000), and myelin basic protein (MBP; Cell Signaling Technology, 78896; 1:1,000). After washing, membranes were incubated with horseradish-peroxidase-conjugated secondary antibody (Cell Signaling Technology, 7074; 1:5,000) for 1 h at room temperature. Protein bands were visualized using enhanced chemiluminescence reagent (Thermo Fisher Scientific, 32106) and imaged on a ChemiDoc MP system (Bio-Rad). Band intensities were quantified using ImageJ or Image Lab software and normalized to β-actin (Cell Signaling Technology, 4970).

### RT-qPCR analysis of Foxp3 mRNA expression

Total RNA was extracted using TRIzol reagent (Invitrogen, 15596026), and RNA purity was assessed with a NanoDrop 2000 spectrophotometer (Thermo Fisher Scientific). One microgram of RNA was reverse-transcribed using the PrimeScript RT Reagent Kit (Takara, RR037A). Quantitative polymerase chain reaction (qPCR) was performed using TB Green Premix Ex Taq II (Takara, RR820A) on a QuantStudio 6 Flex system (Applied Biosystems), with all reactions run in triplicate. Primer sequences were as follows: Foxp3, 5′-CACCTATGCCACCCTTATCCG-3′ (forward) and 5′-CATGCGAGTAAACCAATGGTAGA-3′ (reverse); β-actin, 5′-GTGACGTTGACATCCGTAAAGA-3′ (forward) and 5′-GCCGGACTCATCGTACTCC-3′ (reverse). Relative expression levels were calculated using the 2^−ΔΔCt^ method.

### CFSE labeling of CD8^+^ T cells and construction of Treg cell coculture system

Primary mouse CD8^+^ T cells were isolated by negative selection using the CD8a^+^ T Cell Isolation Kit (Miltenyi Biotec, 130-104-075) and labeled with the CellTrace CFSE Cell Proliferation Kit (Thermo Fisher Scientific, C34554) at a final concentration of 5 μM. Cells were incubated at 37 °C for 10 min, and staining was terminated using complete culture medium. Treg cells were isolated using the CD4^+^CD25^+^ Treg Cell Isolation Kit (Miltenyi Biotec, 130-091-041) and cocultured with carboxyfluorescein diacetate succinimidyl ester (CFSE)-labeled CD8^+^ T cells at a 1:1 ratio. The following groups were established: control, LPS (100 ng/ml; Sigma-Aldrich, L2630), LPS + MOF@PI (50 μg/ml), LPS + free IL-2 (10 ng/ml; PeproTech, 200-02), LPS + MOF@PI-IL2 (50 μg/ml), and LPS + MOF@PI-IL2 (50 μg/ml) + siRNA-mediated Foxp3 KD (siFoxp3). Cocultures were incubated at 37 °C in 5% CO_2_ for 72 h. At least 50,000 events were acquired per sample using a BD LSRFortessa flow cytometer (BD Biosciences), and the proliferation index was calculated using FlowJo v10.

### Transwell-based assessment of Treg-cell-mediated suppression of CD8^+^ T cell activation

Transwell coculture assays were performed using 0.4-μm pore-size Millicell inserts (Millipore, PICM01250). Treg cells were seeded in the upper chamber, and unlabeled CD8^+^ T cells were placed in the lower chamber to prevent direct cell–cell contact. After 48 h, cells from the lower chamber were collected and stained with anti-CD69–phycoerythrin antibody (BioLegend, 104508; 1:200) for 30 min at 4 °C in the dark. Samples were analyzed on a BD LSRFortessa flow cytometer, and CD69 positivity was quantified using FlowJo v10.

### ELISA analysis

After 48 h of treatment, supernatants from Treg cell cultures were collected and clarified by centrifugation at 300*g* for 5 min at 4 °C. Concentrations of IL-10, TGF-β1, IL-6, and tumor necrosis factor-α (TNF-α) were measured using mouse IL-10 (R&D Systems, DY417), TGF-β1 (R&D Systems, DY1679), IL-6 (R&D Systems, DY406), and TNF-α (R&D Systems, DY410) ELISA kits, respectively. For TGF-β1 measurement, samples were acid-activated before analysis. In supernatants from CD8^+^ T cell cocultures, interferon-γ (IFN-γ) and granzyme B were measured using mouse IFN-γ (R&D Systems, DY485) and mouse granzyme B (R&D Systems, DY1865) ELISA kits. Samples were diluted 1:2 and processed according to the manufacturers’ instructions. Absorbance was measured at 450 nm using a SpectraMax iD3 microplate reader (Molecular Devices).

### Microscopic imaging and image recording of the coculture system

Images from CFSE dilution assays were acquired using a Leica DMi8 microscope (Leica Microsystems) with 488-nm excitation. Image resolution was set to 1,024 × 1,024 pixels, and all exposure settings were kept constant across groups. Raw images were exported using LAS X software without enhancement or cropping.

### Construction of a dorsal root ganglion neuron injury model and establishment of the Treg cell coculture system

Lumbar dorsal root ganglion (DRG) neurons were isolated from mice via enzymatic dissociation and processed using the Adult Mouse DRG Neuron Dissociation Kit (BrainBits, DRG-KIT). Cells were seeded onto glass coverslips precoated with poly-d-lysine (Sigma-Aldrich, P6407) and laminin (Thermo Fisher Scientific, 23017015). Neuronal injury was induced with LPS (Sigma-Aldrich, L2630) at a final concentration of 1 μg/ml for 24 h. Treg cells were isolated using the CD4^+^CD25^+^ Treg Cell Isolation Kit (Miltenyi Biotec, 130-091-041) and added to the coculture system at a DRG:Treg cell ratio of 3:1. Cells were then treated with MOF@PI (50 μg/ml), free IL-2 (PeproTech, 200-02; 10 ng/ml), or MOF@PI-IL2 (50 μg/ml) for 48 h before subsequent analyses.

### Whole-cell patch-clamp recording of DRG neuron action potentials

Action potentials were recorded using an Axon Patch 200B amplifier (Molecular Devices) and a Digidata 1550B analog–digital converter. Recording electrodes were pulled from borosilicate glass capillaries (Sutter Instrument, BF150-86-10) with a resistance of 3 to 5 MΩ and filled with an internal solution containing 140 mM K-gluconate, 10 mM Hepes, 4 mM Mg–adenosine triphosphate, and 0.3 mM Na–guanosine triphosphate (pH 7.25). Action potential firing frequency and amplitude were recorded under constant current injection (+20 to +80 pA). Data acquisition and analysis were performed using pCLAMP 10 software. No fewer than 50 neurons were recorded per group.

### Ca^2+^ imaging analysis of DRG neurons

Intracellular calcium imaging was performed using Fluo-4 acetoxymethyl ester (Thermo Fisher Scientific, F14201) at a final concentration of 5 μM. Cells were incubated at 37 °C for 30 min and then imaged in real time using a Leica DMi8 microscope with 488-nm excitation. Time-lapse images were acquired at 1 frame/s for 5 min. Peak Ca^2+^ amplitude and event frequency were quantified using LAS X software, with at least 50 neurons analyzed per group.

### Immunofluorescence staining

Cell samples were fixed with 4% paraformaldehyde for 15 min, permeabilized with 0.2% Triton X-100 for 10 min, and blocked with 5% bovine serum albumin at room temperature for 1 h. Growth-associated protein 43 (GAP43) antibody (Abcam, ab16053; 1:300) and neurofilament 200 (NF200) antibody (Sigma-Aldrich, N0142; 1:400) were then added and incubated overnight at 4 °C. For tissue sections, heat-induced antigen retrieval was performed in citrate buffer (pH 6.0), followed by blocking with 5% bovine serum albumin and overnight incubation at 4 °C with CD68 antibody (Abcam, ab125212; 1:300) and inducible nitric oxide synthase (iNOS) antibody (Abcam, ab15323; 1:300). The following day, Alexa Fluor 488 or 594 secondary antibodies (Thermo Fisher Scientific, A-11008/A-11012; 1:500) were applied, and nuclei were counterstained with DAPI (Sigma-Aldrich, D9542). Imaging was conducted using a Zeiss LSM 880 confocal microscope with consistent excitation and exposure parameters across samples. Fluorescence intensity of GAP43 and NF200, as well as the signal area of CD68^+^ and iNOS^+^ staining, was quantified in ImageJ using a unified thresholding strategy.

### Axonal tracing and neuronal morphology quantification

Neuronal morphology was assessed from high-resolution images obtained after βIII-tubulin immunostaining (Cell Signaling Technology, 5568; 1:500). Axonal skeletons were reconstructed using the Simple Neurite Tracer plugin in ImageJ. Average axon length, branch number, and Sholl analysis parameters were quantified under a uniform threshold setting. At least 50 neurons were analyzed per group.

### Establishment of the DPN model

Eight-week-old male C57BL/6J mice were obtained from Vital River/Charles River Laboratory (China). After 1 week of acclimatization under specific-pathogen-free conditions, diabetes was induced by intraperitoneal injection of streptozotocin (Sigma-Aldrich, S0130) dissolved in citrate buffer (pH 4.5) at 50 mg/kg for 5 consecutive days after 4 h of fasting. Blood glucose levels were monitored regularly, and mice with glucose levels >16.7 mmol/l were considered diabetic. DPN phenotypes were assessed 4 weeks after streptozotocin injection.

Mice were subsequently divided into 5 groups (*n* = 10 per group): the sham group (no treatment), the DPN + NS group (treated with normal saline), the DPN + free IL-2 group (treated with free IL-2), the DPN + MOF@PI-IL2 group (treated with MOF@PI-IL2 nanovesicles), and the DPN + MOF@PI-IL2 + Foxp3 KD group (received Foxp3 KD followed by treatment with MOF@PI-IL2 nanovesicles).

Foxp3 KD was achieved using an adeno-associated virus (AAV) vector encoding Foxp3 short hairpin RNA (shRNA) (packaged as AAV–Foxp3 shRNA; 1 × 10^11^ viral particles, tail vein injection). Nanocarrier formulations were administered via tail vein injection every 3 d for 2 weeks at an IL-2-equivalent dose of 2 μg per mouse per injection and a MOF@PI dose of 5 mg/kg. All formulations were prepared in sterile PBS and filtered through a 0.22-μm membrane before injection. Mice were maintained under specific-pathogen-free conditions, and body weight, blood glucose, and behavioral changes were monitored throughout the study. All procedures were approved by the institutional animal ethics committee, and all in vivo experiments were performed in a blinded manner.

### In vivo imaging system of Cy5.5-labeled material distribution

Cyanine 5.5 (Cy5.5)-labeled free IL-2, MOF@PI, and MOF@PI-IL2 were prepared by reacting each formulation with *N*-hydroxysuccinimide–Cy5.5 dye (Lumiprobe, 15320). Free dye was removed using a Sephadex G-25 column (GE Healthcare), and the labeled materials were administered by tail vein injection (100 μl per mouse) at equivalent fluorescence intensity. In vivo fluorescence distribution was monitored using an in vivo imaging system (IVIS) spectrum imaging system (PerkinElmer) with fixed acquisition settings. Fluorescence intensity in the sciatic nerve region was quantified using Living Image software.

### DHE fluorescence staining for assessing oxidative stress in sciatic nerves

Sciatic nerve sections (7 μm) were fixed in 95% ethanol and incubated with 10 μM dihydroethidium (DHE; Invitrogen, D11347) in the dark for 30 min. After washing with PBS, fluorescence images were acquired using a Leica DMi8 microscope under identical exposure settings. Fluorescence intensity was quantified in ImageJ using integrated density.

### Assessment of lipid peroxidation and antioxidant enzyme activities (malondialdehyde, superoxide dismutase, and catalase)

Sciatic nerve homogenates were analyzed using commercial assay kits for malondialdehyde (MDA; Beyotime, S0131), superoxide dismutase (SOD; Beyotime, S0101), and catalase (CAT; Solarbio, BC0205). Absorbance was measured at 532, 450, and 405 nm, respectively, using a BioTek Synergy H1 microplate reader, and values were calculated from standard curves.

### Tissue staining

Sciatic nerves were fixed in 4% paraformaldehyde and sectioned into 7-μm paraffin slices. For Luxol Fast Blue (LFB) staining, sections were incubated in 0.1% LFB solution (Sigma-Aldrich, S3382) at 60 °C for 18 h, followed by differentiation with lithium carbonate and 70% ethanol until gray matter appeared lighter and white matter retained stable blue staining. Nuclei were counterstained with cresyl violet, and images were acquired using a Leica DM4000 microscope. The myelin area was quantified using the Area Fraction module in ImageJ.

Hematoxylin and eosin (H&E) staining was performed according to the standard protocol provided with the H&E Staining Kit (Baso Diagnostics, BA4099). Inflammatory infiltration area was quantified in ImageJ and normalized to the total tissue area.

Masson’s trichrome staining was performed using a commercial kit (Solarbio, G1340). Collagen-positive areas were identified after color separation with the Color Deconvolution plugin in ImageJ and quantified using a uniform threshold.

### Nerve conduction velocity measurement

Mice were anesthetized with 1.5% isoflurane and placed on a thermostatically controlled platform. Rectal temperature was maintained at 36.5 to 37.5 °C using a feedback-controlled heating system. Electrophysiological recordings were performed using a PowerLab data acquisition system with an electrophysiology amplifier module (ADInstruments, Australia). Disposable needle electrodes were placed at the proximal and distal sites of the left sciatic nerve for stimulation. The recording electrode was inserted into the ipsilateral gastrocnemius muscle, and the reference electrode was placed subcutaneously near the tendon insertion. Stimulation was delivered as monophasic rectangular pulses (2 to 5 mA, 0.1 ms, 1 Hz) until stable compound muscle action potentials were obtained. Nerve conduction velocity (NCV) was calculated as the distance between the 2 stimulation sites divided by the difference in latency.

### Hot plate test

Mice were habituated to the apparatus for 2 consecutive days before testing by placing them on the inactive surface for 5 min daily. For formal testing, a hot plate analgesia meter (Ugo Basile, DS37) was maintained at 52 ± 0.2 °C. Each mouse was placed at the center of the heated surface, and the latency to the first nociceptive response, including paw licking, paw lifting, or jumping, was recorded. A cutoff time of 30 s was applied to prevent tissue injury. Each mouse was tested 3 times at 15-min intervals, and the mean latency was used as the thermal pain threshold.

### Von Frey mechanical threshold test

Mechanical sensitivity was assessed using a dynamic plantar aesthesiometer (Ugo Basile, 37450). Mice were placed individually in transparent enclosures on a wire-mesh platform and allowed to acclimate for 30 min. The probe was applied vertically to the mid-plantar surface of the hind paw with a linearly increasing force. The maximum force was set at 10 g with a ramp rate of 0.25 g/s. The force required to elicit a clear paw withdrawal was recorded as the mechanical threshold.

### Toe spread and pinch reflex tests

Sensorimotor reflexes were assessed at least 24 h apart from other behavioral tests. For the toe spread test, mice were gently suspended by the base of the tail, and images of the hind paws were captured from a posterior view. Toe spread angle was measured in ImageJ, and the mean value from both hind limbs was calculated for each mouse.

For the pinch reflex test, gentle pressure was applied to the skin between the hind toes using fine forceps to elicit a flexion response. Reflexes were scored on a 0 to 3 scale as follows: 0, no response; 1, slight flexion; 2, marked and sustained flexion; and 3, rapid, forceful flexion with trunk retraction. Each mouse was evaluated independently by 2 blinded observers, and discrepant scores were reviewed until consensus was reached.

### Open field test

Anxiety-like behavior and spontaneous locomotor activity were assessed in an open field apparatus (Med Associates; 50 × 50 × 40 cm). Mice were acclimated to the testing room for 30 min before the experiment. During testing, illumination was maintained at 20 to 30 lx. Each mouse was placed in the center of the arena and allowed to explore freely for 10 min. Movements were recorded by an overhead camera and analyzed using the EthoVision XT system (Noldus).

### Y-maze test

Spatial working memory was evaluated using a Y-maze with 3 arms positioned at 120° angles (35 cm × 10 cm × 15 cm each). Mice were acclimated to the testing room for 30 min and then allowed to explore the maze freely for 8 min. Behavior was recorded using an overhead camera and analyzed with EthoVision XT. An arm entry was defined as the entry of both forelimbs and the front half of the body into one arm. Consecutive entries into 3 different arms were counted as a spontaneous alternation. The maze was cleaned with 70% ethanol between trials. All behavioral analyses were performed in a blinded manner.

### RNA extraction and library preparation

Sciatic nerve tissues were lysed immediately after collection in TRIzol reagent (Invitrogen, 15596026) for total RNA extraction. After phase separation with chloroform (Sigma-Aldrich, C2432), RNA was precipitated with isopropanol (Sigma-Aldrich, I9516) and washed with ribonuclease-free 75% ethanol. RNA purity was assessed using a NanoDrop One spectrophotometer (Thermo Fisher Scientific), and RNA integrity was evaluated using an Agilent 2100 Bioanalyzer (Agilent Technologies). Only samples with an absorbance at 260/280 nm ratio of 1.95 to 2.05 and an RNA integrity number of ≥8.0 were used for library construction. Strand-specific libraries were prepared using the NEBNext Ultra II RNA Library Prep Kit (New England Biolabs, E7770), including mRNA enrichment, fragmentation, cDNA synthesis, end repair, adaptor ligation, and PCR amplification. Final libraries were quantified using a Qubit 4 fluorometer (Thermo Fisher Scientific), and fragment size distribution was verified using the Agilent Bioanalyzer.

### RNA-seq

Libraries that passed quality control were sequenced on the NovaSeq 6000 platform (Illumina) using the NovaSeq 6000 S4 Reagent Kit (Illumina, 20027466) in paired-end 150-bp mode. Raw data output was at least 6.0 Gb per sample. Binary base call (BCL) files were converted to FASTQ format using bcl2fastq software (v2.20, Illumina) for downstream analysis.

### Quality control and filtering of sequencing data

Raw FASTQ files were first assessed using FastQC (v0.11.9) for base quality, Q20/Q30 scores, GC content, and adaptor contamination. Reads were then filtered using Trimmomatic (v0.39) with the following parameters: ILLUMINACLIP:2:30:10, LEADING:3, TRAILING:3, SLIDINGWINDOW:4:15, and MINLEN:50. After filtering, all samples showed Q30 scores above 93% and clean read retention rates above 92%.

### Sequence alignment and gene quantification

Clean reads were aligned to the mouse reference genome GRCm38/mm10 (GENCODE vM25) using STAR (v2.7.10a) with the parameters runThreadN = 16, outFilterMismatchNmax = 2, and alignIntronMax = 500,000. Mapping rates ranged from 91% to 95%. Gene-level read counts were generated using featureCounts (v2.0.1) with the parameters -t exon, -g gene_id, -s 0, and -T 16.

### Differential expression analysis

Differential gene expression analysis was performed using DESeq2 (v1.38.0). Count data were normalized by size factors, and differential expression was tested using a negative binomial generalized linear model. Differentially expressed genes (DEGs) were defined as those with |log_2_ fold change| (|log_2_FC|) ≥ 1 and *P* < 0.05. Volcano plots and heatmaps were generated using EnhancedVolcano (v1.16.0) and pheatmap (v1.0.12), respectively.

### Immune infiltration analysis (CIBERSORT)

Immune cell composition in sciatic nerve tissue was estimated using Cell type Identification By Estimating Relative Subsets Of RNA Transcripts (CIBERSORT). The expression matrix was first converted to transcripts per million (TPM) values and then analyzed using the ν-support vector regression model with 1,000 permutations. Relative proportions of 22 immune cell types were obtained. Differences in Treg cell, T helper 1 cell (Th1 cell), Th17 cell, and M1 macrophage infiltration among groups were analyzed using the Wilcoxon rank sum test. Bar plots and box plots were generated using ggplot2 (v3.4.0).

### Gene Ontology and Kyoto Encyclopedia of Genes and Genomes functional enrichment analysis

To systematically investigate the biological processes, cellular localizations, and signaling pathways associated with DEGs, Gene Ontology (GO) and Kyoto Encyclopedia of Genes and Genomes (KEGG) enrichment analyses were conducted using the Xiantao Academic Database (https://www.xiantaozi.com/). GO analysis encompassed the biological processes, cellular component, and molecular function categories, and KEGG analysis was restricted to the mouse genome (species = “mmu”). The statistical threshold was set at *P*.adjust < 0.05, with a minimum gene set size of 10 and a maximum of 500.

### Weighted gene coexpression network analysis construction

Weighted gene coexpression network analysis (WGCNA) was performed using the WGCNA package (v1.71). Gene expression data were normalized as log_2_(TPM + 1), and the top 5,000 genes ranked by median absolute deviation were selected. A scale-free coexpression network was constructed, and modules were identified using hierarchical clustering with dynamic tree cutting. The minimum module size was set to 100, and modules with a similarity of >0.4 were merged. Module eigengenes were correlated with immune infiltration profiles from CIBERSORT using Pearson correlation analysis. Modules associated with Treg-cell-related genes, including Foxp3, Il2ra, Ctla4, and Stat5b, were further analyzed.

### Metabolomic data acquisition and preprocessing

Untargeted serum metabolomics was performed using a Q Exactive Orbitrap mass spectrometer coupled to a Vanquish ultrahigh-performance liquid chromatography system (Thermo Fisher Scientific). Chromatographic separation was achieved on a Hypersil GOLD C18 column (100 × 2.1 mm, 1.9 μm; Thermo Fisher Scientific, 25003-102130). The mobile phases consisted of 0.1% formic acid in water and 0.1% formic acid in acetonitrile, with a flow rate of 0.30 ml/min. Data were acquired in both positive and negative ionization modes at a resolution of 70,000. Peak detection and alignment were performed using XCMS (v3.18.0), and isotopes and adducts were annotated using CAMERA (v1.54.0). Peak areas were normalized by Pareto scaling and log_10_ transformation. Differential metabolites were identified using limma (v3.52) with thresholds of |log_2_FC| ≥ 1 and *P* < 0.05.

### Integrated metabolomic–RNA-seq correlation analysis

To investigate the relationship between metabolic alterations and Treg-cell-associated transcriptional changes, Spearman correlation analysis was performed between differential metabolites and the expression levels of representative Treg-cell-related genes (including Foxp3, Il2ra, Stat5b, and Ctla4) and proinflammatory genes (including Ifng and Il17a) across individual samples. Metabolite abundance was normalized by Pareto scaling, followed by log_10_ transformation, whereas gene expression was represented as log_2_(TPM + 1). Correlation coefficients and *P* values were calculated using the cor.test function in R (v4.2.2). Scatter plots were generated using ggplot2 (v3.4.0), with metabolite abundance plotted against gene expression for each sample. Linear regression lines and 95% confidence intervals were overlaid for visualization. Comparisons were displayed separately for the Sham versus DPN + NS and DPN + NS versus DPN + MOF@PI-IL2 groups using uniform axis scales.

### Statistical analysis

Data analysis and visualization were primarily conducted using GraphPad Prism 9.0 and R software (version 4.2.2), incorporating packages such as tidyverse, limma, DESeq2, ggplot2, and pheatmap. Rigorous statistical tests and modeling approaches were used throughout this study. In vitro experiments were typically performed with at least 3 independent biological replicates to ensure data reliability and consistency. For animal experiments, each group consisted of 10 mice. Normality of the data was assessed using the Shapiro–Wilk test, while homogeneity of variance was evaluated via Levene’s test to determine the appropriate statistical approach. For comparisons between 2 groups, a 2-tailed *t* test was used when data followed a normal distribution; otherwise, the Mann–Whitney *U* test was applied. For comparisons involving more than 2 groups, 1-way or 2-way analysis of variance (ANOVA) was performed as appropriate, followed by post hoc tests, including Tukey’s or Bonferroni corrections. For time-series or repeated-measures data, a repeated-measures ANOVA was used. A *P* value of less than 0.05 was considered statistically significant.

## Results

### Successful construction and systematic validation of structurally uniform MOF@PI-IL2 nanovesicles

**Fig. 1.**
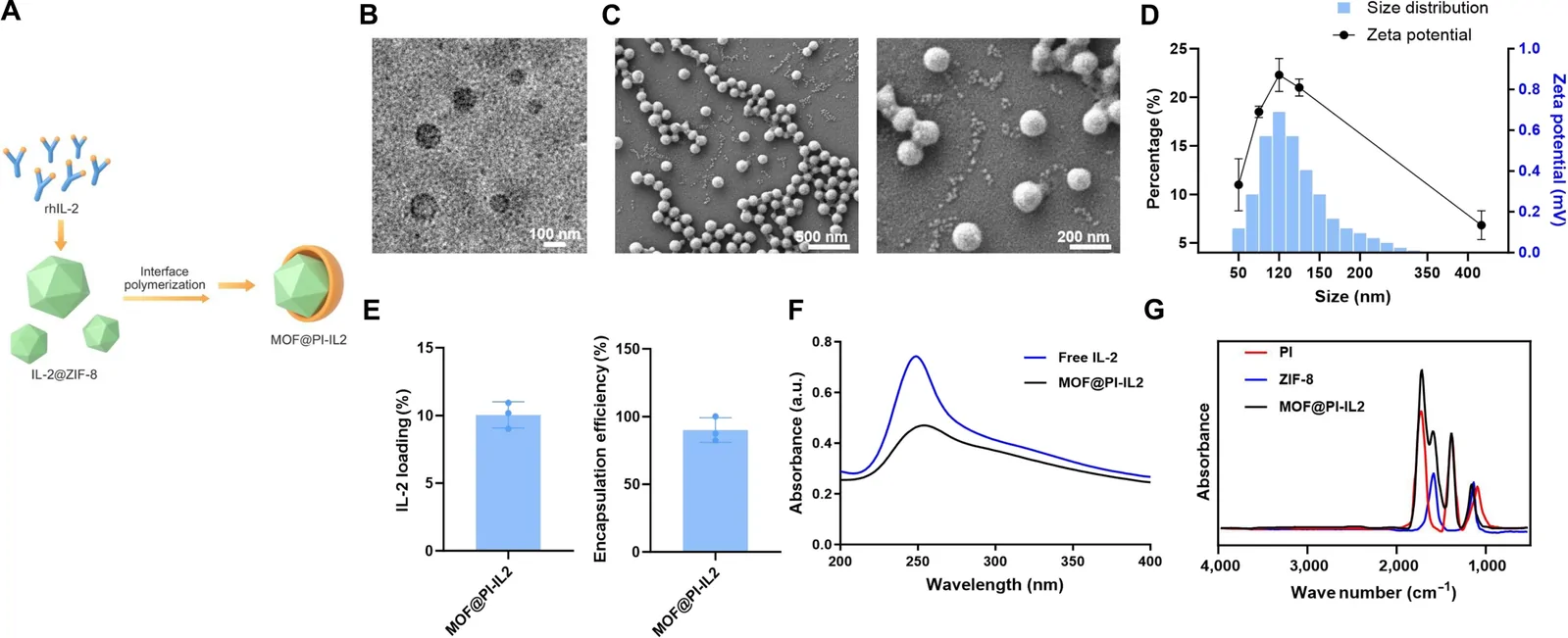
Structural construction and comprehensive physicochemical characterization of polyimide (PI)-coated zeolitic imidazolate framework-8 (ZIP-8) core–shell nanovesicles loaded with interleukin-2 (IL-2) (MOF@PI-IL2) nanovesicles. (A) Schematic illustration showing the biomineralization of IL-2 to form IL-2-loaded ZIF-8 (IL-2@ZIF-8), followed by PI cross-linking to generate the core–shell structured MOF@PI-IL2 nanovesicles. (B) Transmission electron microscopy (TEM) images depicting nanovesicle morphology. Scale bar, 100 nm. (C) Scanning electron microscopy (SEM) images showing surface morphology and dispersion of the nanovesicles. Scale bars, 500 and 200 nm. (D) Dynamic light scattering (DLS) analysis of hydrodynamic diameter and zeta potential. (E) Enzyme-linked immunosorbent assay (ELISA)-based quantification of IL-2 loading and encapsulation efficiency. (F) Ultraviolet–visible (UV–Vis) spectra showing characteristic IL-2 absorption peaks. (G) Fourier transform infrared (FTIR) spectra showing PI imide peaks and ZIF-8 framework features. All experiments were repeated at least 3 times.

To develop a nanoplatform capable of stable IL-2 delivery and pH-responsive release in an inflammatory acidic microenvironment, we first synthesized IL-2@ZIF-8 nanoparticles by biomineralization in the presence of rhIL-2, enabling efficient encapsulation of IL-2 within the MOF core. A biocompatible PI precursor was then introduced to form a dense ultrathin shell on the IL-2@ZIF-8 surface through interfacial condensation and cross-linking, yielding MOF@PI-IL2 nanovesicles with improved structural stability and enhanced protection of the protein cargo (Fig. 1A).

TEM showed that IL-2@ZIF-8 displayed the characteristic rhombic morphology of ZIF-8 nanocrystals. After PI coating, the particles retained their overall structure while exhibiting a continuous and uniform shell layer, without obvious collapse (Fig. 1B). SEM further demonstrated that MOF@PI-IL2 particles were uniformly dispersed and exhibited a smooth, compact, and near-spherical morphology, with no evident aggregation or structural disruption (Fig. 1C).

Previous studies have shown that the particle size of ZIF-8-based nanoparticles can be tuned within a range of approximately 30 to 150 nm and that a unimodal, narrow distribution is an important indicator of colloidal stability for this class of nanocarriers [28]. DLS analysis showed that MOF@PI-IL2 particles were mainly distributed within the range of 100 to 150 nm with a unimodal profile, and the dominant population was centered at approximately 120 nm, accounting for nearly 22% of the total distribution. The zeta potential profile showed a gradual decrease from approximately 20 to 25 mV to near-neutral values, indicating stable surface-charge characteristics (Fig. 1D). ELISA-based quantification further revealed that IL-2 loading was approximately 10%, while encapsulation efficiency reached about 90%, indicating efficient incorporation of IL-2 during particle fabrication (Fig. 1E).

Spectroscopic analyses further confirmed the compositional integrity of the nanovesicles. UV–Vis spectra showed that the characteristic absorption peaks of IL-2 were retained after encapsulation (Fig. 1F). FTIR spectra showed clear imide stretching peaks of PI (approximately 1,720 and 1,380 cm^−1^), while the Zn–N stretching band of the ZIF-8 framework remained preserved, confirming successful integration of IL-2, PI, and MOF without disruption of the crystal framework (Fig. 1G).

Taken together, these results demonstrate the successful fabrication of MOF@PI-IL2 nanovesicles with a well-defined core–shell structure, uniform particle morphology, and efficient IL-2 loading.

### MOF@PI-IL2 exhibits physiological stability and weak-acid responsiveness adapted to the inflammatory microenvironment of DPN

To evaluate whether MOF@PI-IL2 possesses delivery properties suited to the inflammatory microenvironment of DPN, we assessed its colloidal stability, structural integrity, shell responsiveness, and release behavior under different pH conditions.

**Fig. 2.**
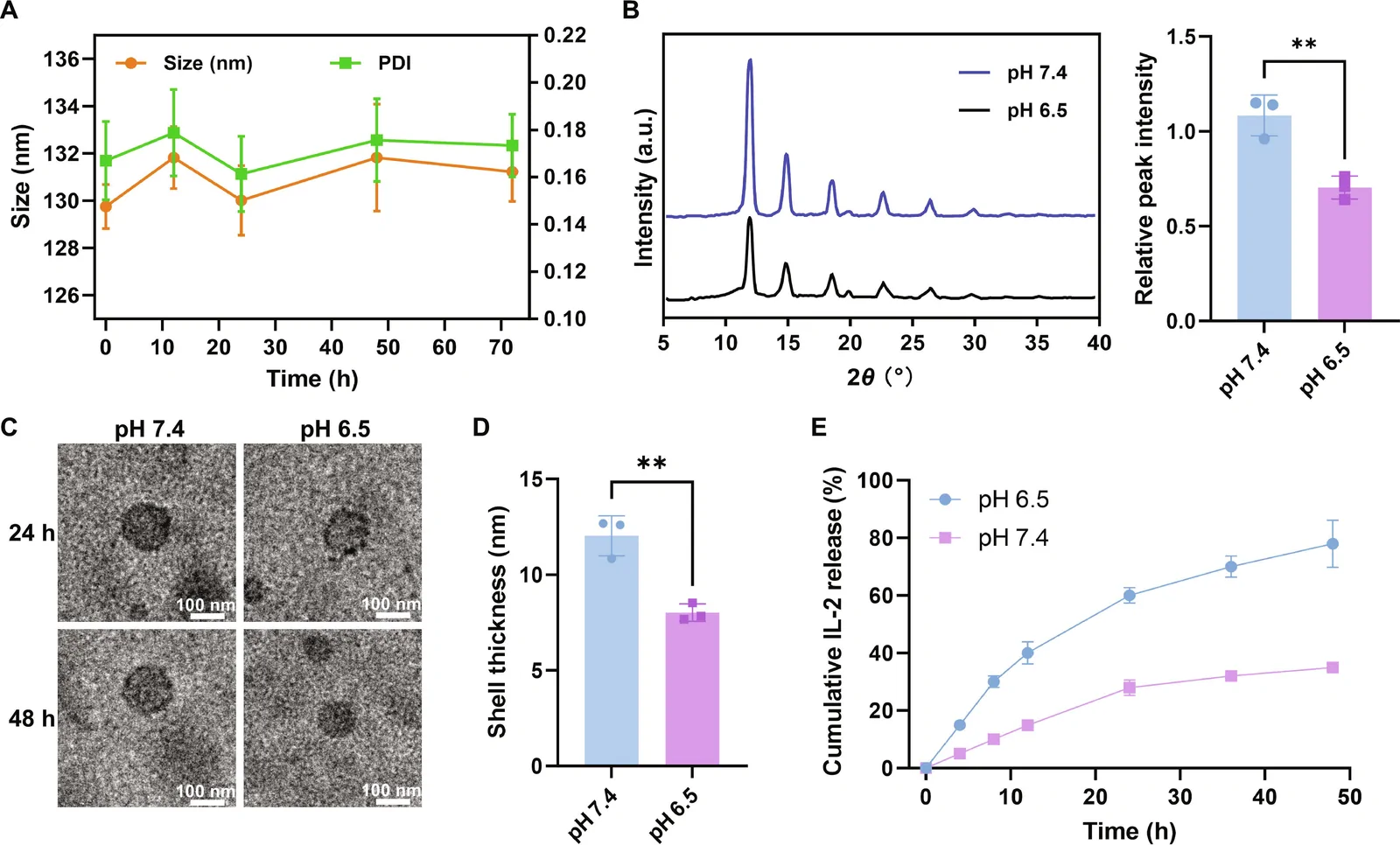
Multidimensional stability and pH-responsive interleukin-2 (IL-2) release profile of polyimide (PI)-coated zeolitic imidazolate framework-8 core–shell nanovesicles loaded with IL-2 (MOF@PI-IL2). (A) Dynamic light scattering (DLS) analysis of particle size and polydispersity index (PDI) of MOF@PI-IL2 over 72 h under pH 7.4 conditions. (B) X-ray diffraction analysis of MOF@PI structural features after incubation under different pH conditions. (C) Transmission electron microscopy (TEM) images of nanovesicle shell morphology under pH 7.4 and pH 6.5. Scale bars, 100 nm. (D) Quantification of PI shell thickness under different pH conditions based on TEM images (*n* = 50 particles). (E) Cumulative IL-2 release profiles of MOF@PI-IL2 at pH 7.4 and pH 6.5, measured by enzyme-linked immunosorbent assay (ELISA). All experiments were performed in triplicate. **P < 0.01.

Under simulated physiological conditions (pH 7.4), DLS measurements showed that particle size remained stable within the range of 120 to 150 nm over 72 h, with fluctuations below 5% and a consistently low PDI (PDI < 0.2) (Fig. 2A). These findings indicate good colloidal stability of MOF@PI-IL2 under physiological conditions.

X-ray diffraction analysis was then performed to examine pH-dependent changes in core crystallinity. After incubation at pH 7.4 for 48 h, MOF@PI-IL2 retained the characteristic diffraction peaks of ZIF-8. In contrast, samples incubated at pH 6.5 showed partial peak attenuation and slight broadening (Fig. 2B), suggesting partial loosening of the ZIF-8 crystal structure under mildly acidic conditions.

Subsequently, we conducted a time-course TEM analysis to investigate structural changes in the PI shell under different pH conditions. At pH 7.4, the shell remained continuous and compact over 24-48 h. In contrast, under pH 6.5, the PI shell became mildly loosened, with local shell thickness decreasing by about 0.3-fold (*P* < 0.05) (Fig. 2C). Quantification based on 50 particles further confirmed that the mildly acidic environment significantly relaxed the PI shell and reduced its thickness by about 0.3-fold (*P* < 0.05), thereby increasing its permeability (Fig. 2D).

Functionally, we monitored IL-2 release kinetics under varying pH conditions. At pH 6.5, the cumulative release of IL-2 from MOF@PI-IL2 reached nearly 60% within 24 h, markedly higher than the level under pH 7.4, which remained below 30%. Here, the significance of the ~30% release level lies in its clear contrast with the weakly acidic inflammatory condition rather than implying absolute zero leakage (Fig. 2E).

Collectively, these data show that MOF@PI-IL2 remains stable under physiological conditions while exhibiting mild-acid-responsive structural relaxation and enhanced IL-2 release, supporting its suitability as an inflammation-responsive delivery system.

### MOF@PI-IL2 exhibits selective uptake and endocytic transport in Treg cells

To determine whether MOF@PI-IL2 could be efficiently internalized by Treg cells and reach intracellular compartments compatible with IL-2 release, we prepared DiI-labeled nanovesicles and incubated them with primary murine CD4^+^ T cells to assess cellular uptake, subset preference, and intracellular trafficking.

**Fig. 3.**
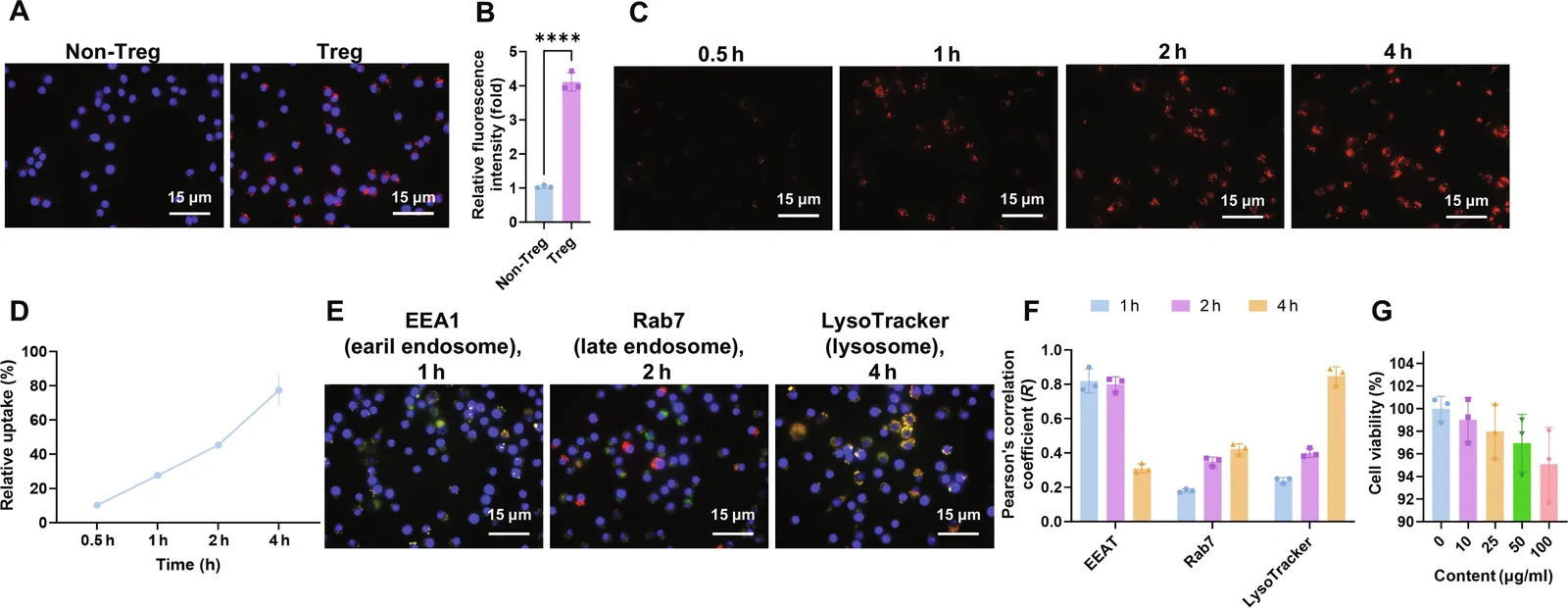
Relative preferential uptake and intracellular trafficking of polyimide-coated zeolitic imidazolate framework-8 core–shell nanovesicles loaded with interleukin-2 (IL-2) (MOF@PI-IL2) in regulatory T cells. (A) Confocal imaging showing the uptake of DiI (1,1′-dioctadecyl-3,3,3′,3′-tetramethylindocarbocyanine perchlorate)-labeled MOF@PI-IL2 (DiI-MOF@PI-IL2) by regulatory T cells (Treg cells). Scale bars, 15 μm. (B) Single-cell fluorescence intensity analysis comparing uptake levels in Treg versus non-Treg cells. (C) Time-lapse confocal imaging of the endocytic process from 0.5 to 4 h. Scale bars, 15 μm. (D) Time-dependent fluorescence intensity curves illustrating endocytic kinetics. (E) Colocalization imaging showing intracellular distribution of MOF@PI-IL2 with endosome antigen-1 (EEA1), Ras-related protein Rab-7a (Rab7), and LysoTracker. Scale bars, 15 μm. (F) Pearson’s correlation analysis quantifying colocalization between MOF@PI-IL2 and endocytosis-related markers. (G) Cell Counting Kit-8 (CCK-8) assay evaluating Treg cell viability after treatment with varying concentrations of MOF@PI-IL2. All experiments were performed in triplicate. ****P < 0.0001.

Confocal imaging showed marked cytoplasmic accumulation of DiI-MOF@PI-IL2 in flow-sorted CD4^+^CD25^+^Foxp3^+^ Treg cells after 4 h of incubation, whereas only weak fluorescence was detected in synchronously sorted CD4^+^CD25^−^ non-Treg cells (Fig. 3A). Quantitative single-cell analysis further showed that the mean fluorescence intensity in Treg cells was approximately 4-fold higher than that in non-Treg cells (*P* < 0.05), indicating preferential uptake by the Treg cell subset rather than absolute selectivity (Fig. 3B).

Time-lapse imaging performed at 0.5, 1, 2, and 4 h revealed that intracellular fluorescence became detectable at 1 h, accumulated as punctate-vesicle-like structures by 2 h, and reached its highest level at 4 h (Fig. 3C). Fitting of fluorescence intensity over time showed a typical saturable uptake profile, consistent with active endocytic internalization (Fig. 3D).

To further elucidate the intracellular trafficking pathway of the nanomaterial, we performed colocalization analyses using EEA1 (early endosome), Rab7 (late endosome), and LysoTracker (lysosome) (Fig. 3E). MOF@PI-IL2 showed clear colocalization with EEA1 at 1 to 2 h, whereas colocalization with LysoTracker increased by about 1.2-fold at 4 h (Fig. 3F), indicating transport along a canonical endosome–lysosome route. This process matches the mildly acidic environment required for IL-2 release and may trigger partial dissociation of the MOF core together with relaxation of the outer shell, thereby favoring controlled intracellular IL-2 release.

Biocompatibility was then evaluated in Treg cells. CCK-8 assays showed that cell viability remained above 95% across the tested concentration range (Fig. 3G).

Fluorescent labeling and confocal imaging showed that MOF@PI-IL2 undergoes relatively preferential uptake in Treg cells and completes intracellular transport via the endosome–lysosome pathway.

### MOF@PI-IL2 reverses LPS-induced Treg cell suppression and amplifies the IL-2/STAT5 signaling pathway

To determine whether MOF@PI-IL2 could reverse inflammation-induced Treg cell suppression, we established an LPS-driven immune dysregulation model and used it for comparative functional analyses.

**Fig. 4.**
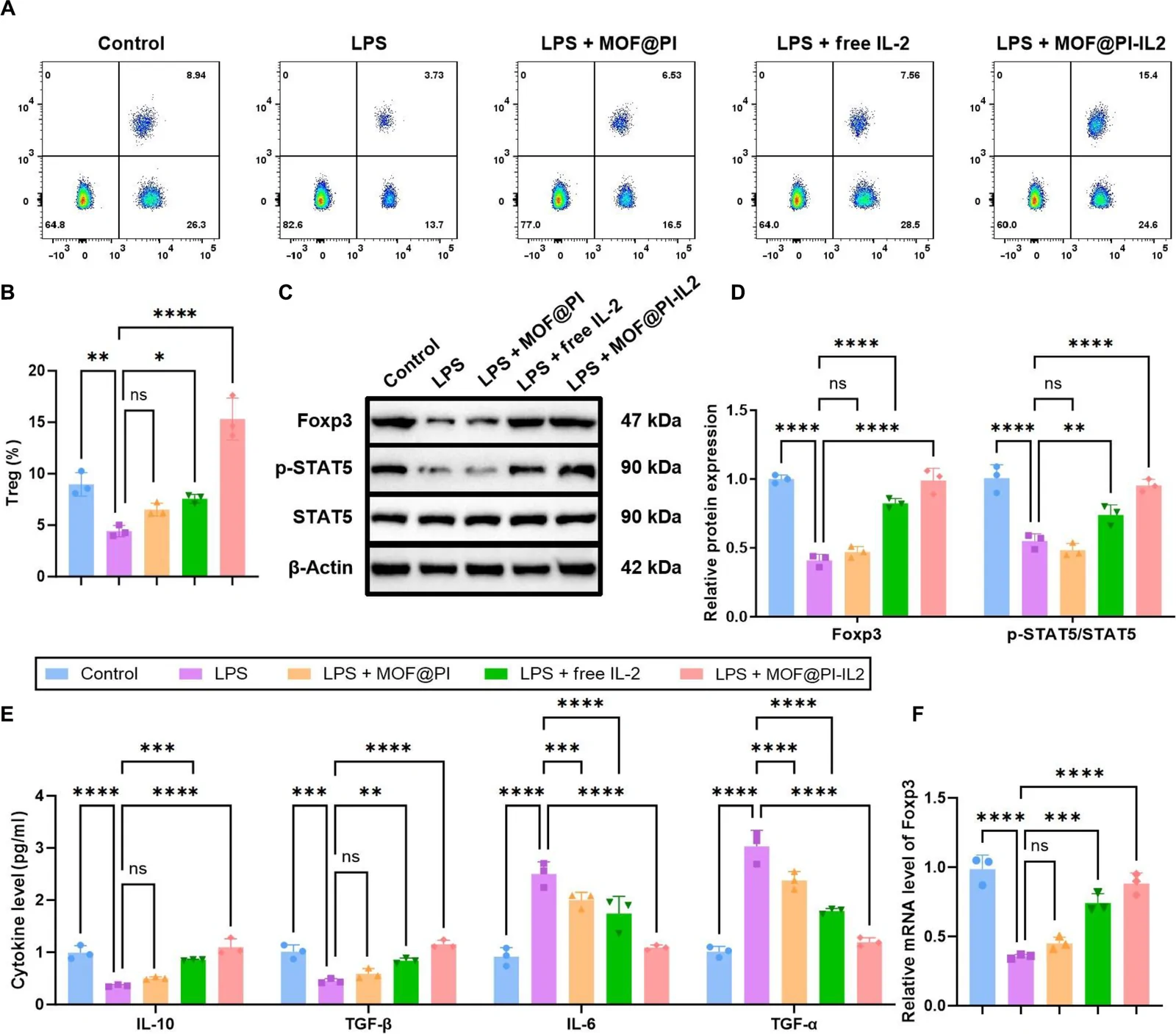
Polyimide-coated zeolitic imidazolate framework-8 core–shell nanovesicles loaded with interleukin-2 (IL-2) (MOF@PI-IL2) reverses lipopolysaccharide (LPS)-induced regulatory T cell (Treg cell) suppression and enhances interleukin-2 (IL-2)/signal transducers and activators of transcription 5 (STAT5) signaling. (A) Flow cytometry analysis of CD4^+^CD25^+^Foxp3^+^ Treg cells. (B) Quantification of Treg cell proportions. (C) Western blot (WB) analysis of phosphorylated STAT5 (p-STAT5)/STAT5 and forkhead box P3 (Foxp3) protein expression. (D) Densitometric quantification of WB bands. (E) Enzyme-linked immunosorbent assay (ELISA) measurement of IL-10, transforming growth factor-β (TGF-β), IL-6, and tumor necrosis factor-α (TNF-α) levels. (F) Reverse transcription quantitative polymerase chain reaction (RT-qPCR) quantification of Foxp3 mRNA expression. All experiments were performed in triplicate. ns, not significant; *P < 0.05; **P < 0.01; ***P < 0.001; ****P < 0.0001.

Flow cytometry analysis showed that LPS markedly reduced the proportion of CD4^+^CD25^+^Foxp3^+^ Treg cells, whereas free IL-2 produced only partial recovery. In contrast, MOF@PI-IL2 significantly increased the Treg cell proportion and restored it within 48 h to a level exceeding baseline (Fig. 4A). Quantitative analysis showed that the Treg cell proportion in the LPS + MOF@PI-IL2 group was approximately 2.1-fold higher than that in the LPS group (*P* < 0.05), highlighting the advantage of sustained IL-2 delivery in expanding Treg cells. Notably, the Treg cell proportion in the LPS + MOF@PI empty-carrier group was 6.53%, which was not significantly different from that in the LPS model group (3.73%, *P* > 0.05) and remained markedly lower than that in the LPS + MOF@PI-IL2 group (15.4%), suggesting that the carrier alone or Zn2^+^ release does not account for Treg cell expansion.

Regarding functional phenotypes, we focused on changes in Foxp3 expression and the IL-2/STAT5 signaling axis. Western blot (WB) analysis revealed that LPS markedly suppressed both p-STAT5 and Foxp3 expression, whereas free IL-2 achieved only partial restoration. In contrast, MOF@PI-IL2 significantly enhanced p-STAT5 levels and concomitantly increased Foxp3 protein expression (Fig. 4C). Quantitative analysis further confirmed significant increases relative to the LPS group (*P* < 0.05), indicating that MOF@PI-IL2 effectively reconstructs the core transcriptional network of Treg cells through sustained IL-2 stimulation. In addition, the LPS + MOF@PI empty-carrier group showed p-STAT5 and Foxp3 levels comparable to those of the LPS model group (*P* > 0.05), confirming that activation of IL-2/STAT5 signaling depends on IL-2 loading (Fig. 4D).

Subsequently, we evaluated the immunoregulatory function of Treg cells. ELISA results showed that LPS significantly reduced IL-10 and TGF-β secretion by approximately 0.6-fold (*P* < 0.05), whereas free IL-2 partially improved this phenotype (*P* < 0.05). By contrast, MOF@PI-IL2 increased IL-10 and TGF-β levels by about 1.2-fold (*P* < 0.05) and simultaneously suppressed proinflammatory cytokines such as IL-6 and TNF-α by about 0.5-fold (*P* < 0.05), indicating clear restoration of the anti-inflammatory effects of Treg cells. In addition, the LPS + MOF@PI empty-carrier group showed no significant differences from the LPS model group (*P* > 0.05), further indicating that the observed recovery depended on IL-2 cargo rather than the carrier itself.

Finally, to determine whether Foxp3 was reestablished at the transcriptional level, we assessed its mRNA abundance. Reverse transcription qPCR (RT-qPCR) results showed that Foxp3 was significantly down-regulated in the LPS group by about 0.7-fold (*P* < 0.05), whereas expression increased by more than 2-fold under LPS + MOF@PI-IL2 treatment relative to the model group (*P* < 0.05) (Fig. 4F), further supporting its ability to remodel the Treg cell gene expression program under inflammatory conditions.

Flow cytometry, WB, and ELISA collectively demonstrated that MOF@PI-IL2 reverses inflammation-induced suppression of Treg cell function, activates the IL-2/STAT5/Foxp3 signaling axis, and reconstructs the anti-inflammatory cytokine network.

### MOF@PI-IL2 enhances Treg-cell-mediated immunosuppression and significantly inhibits aberrant CD8^+^ T cell proliferation and cytotoxic molecule secretion

To determine whether MOF@PI-IL2 enhances Treg cell immunosuppressive capacity following improved phenotype and function, we established an in vitro coculture model using CFSE-labeled CD8^+^ T cells. This system simulated the excessive CD8^+^ T cell activation commonly observed in DPN. Five experimental groups were established: control, LPS, LPS + MOF@PI, LPS + free IL-2, and LPS + MOF@PI-IL2.

First, CD8^+^ T cell proliferation was assessed via CFSE dilution. Under inflammatory stimulation by LPS, CD8^+^ T cells exhibited markedly increased proliferation, reflected by strong dilution of CFSE fluorescence. Compared with Treg cells treated with free IL-2, Treg cells treated with MOF@PI-IL2 showed the strongest inhibition of CD8^+^ T cell proliferation (Fig. S1A). Quantification of the proliferation index showed an approximately 0.5-fold reduction in the MOF@PI-IL2 group (*P* < 0.05), indicating that this platform restores and substantially enhances the proliferation-suppressive function of Treg cells. In addition, the LPS + MOF@PI empty-carrier group showed no significant difference from the LPS model group (*P* > 0.05), supporting that the carrier alone is insufficient to restore Treg-cell-mediated immunosuppression (Fig. S1B).

Next, we assessed changes in key cytotoxic factors, including IFN-γ and granzyme B. ELISA analysis of coculture supernatants revealed that MOF@PI-IL2 treatment significantly reduced secretion of these cytotoxic molecules by CD8^+^ T cells by about 0.5-fold (*P* < 0.05). In contrast, the LPS + MOF@PI empty-carrier group showed IFN-γ and granzyme B levels comparable to those of the LPS model group (*P* > 0.05), with no independent suppressive effect (Fig. S1C). This result indicates that Treg cells activated by MOF@PI-IL2 can effectively weaken the cytotoxic activity of CD8^+^ T cells.

To further assess the regulatory effect on CD8^+^ T cell activation, we used a Transwell system to evaluate CD69 expression. The results demonstrated that, even in the absence of direct cell–cell contact, Treg cells treated with MOF@PI-IL2 significantly reduced CD69 expression in CD8^+^ T cells by about 0.4-fold (*P* < 0.05). In addition, the LPS+MOF@PI empty-carrier group showed no significant difference from the LPS model group (*P* > 0.05), indicating that the empty carrier does not independently regulate the activation state of CD8^+^ T cells (Fig. S1D and E). This finding suggests that the enhanced suppressive effect relies in part on soluble inhibitory mediators rather than entirely on contact-dependent mechanisms.

To test this possibility, we measured IL-10 and TGF-β levels in conditioned medium from Treg cell cultures. These 2 key immunosuppressive mediators were significantly higher in the MOF@PI-IL2 group than in the free IL-2 group, by about 1.8-fold (*P* < 0.05) (Fig. S1F), suggesting that the enhanced immunoregulatory effect of MOF@PI-IL2 depends on an IL-10/TGF-β-dominant anti-inflammatory cytokine network.

CFSE proliferation assays, Transwell coculture, and cytotoxic factor analyses collectively demonstrated that MOF@PI-IL2 enhances Treg-cell-mediated immunosuppression and attenuates CD8^+^ T cell cytotoxic activity.

### MOF@PI-IL2 promotes functional recovery and structural reconstruction of injured DRG neurons

In an LPS-induced DRG neuron injury model, we further evaluated whether Treg cells activated by MOF@PI-IL2 could modulate the functional and structural recovery of damaged peripheral neurons. Coculture experiments were conducted under 5 conditions: control, LPS, LPS + MOF@PI, LPS + free IL-2, and LPS + MOF@PI-IL2.

**Fig. 5.**
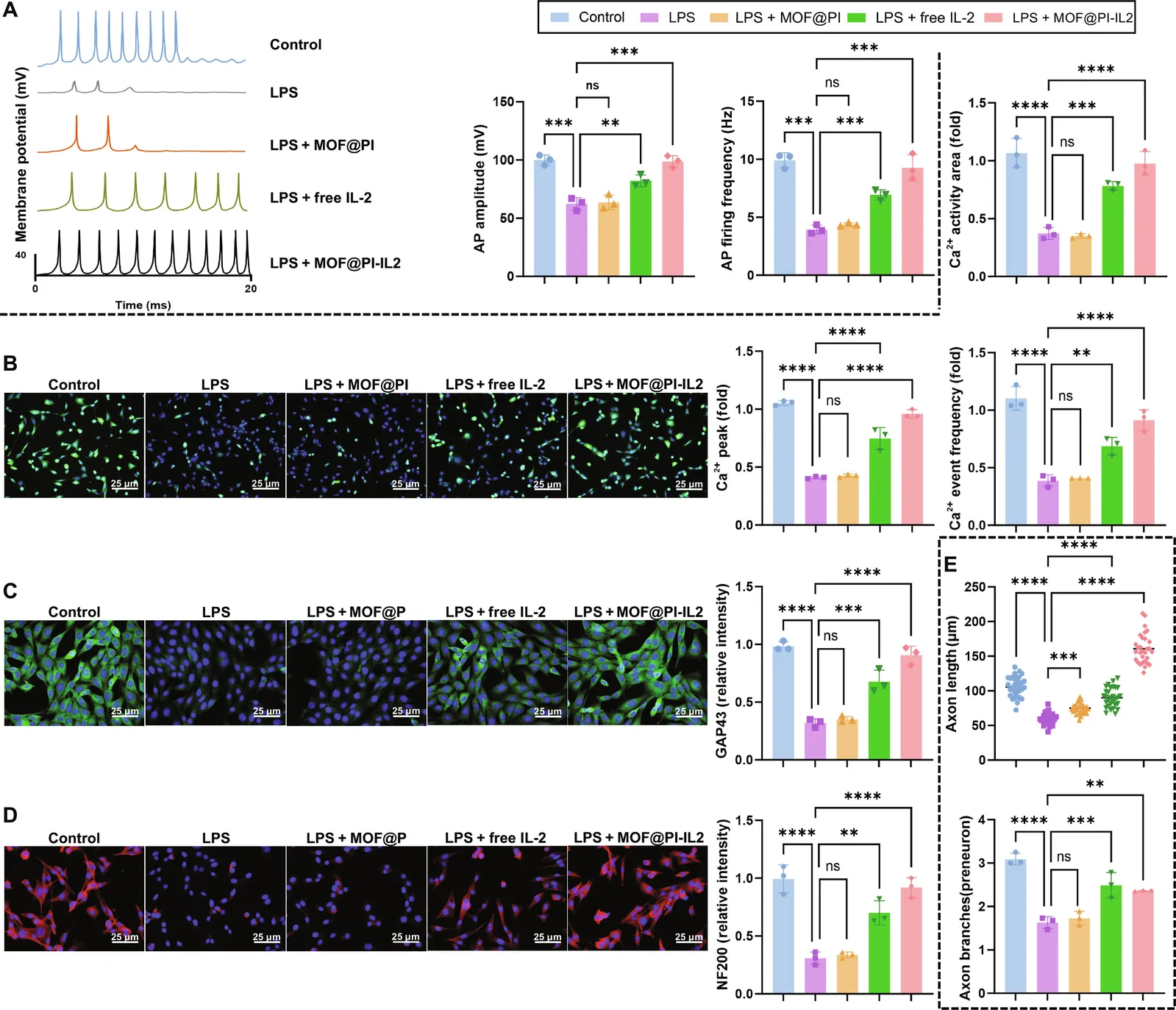
Polyimide-coated zeolitic imidazolate framework-8 core–shell nanovesicles loaded with interleukin-2 (MOF@PI-IL2) promotes functional recovery and structural regeneration of injured dorsal root ganglion (DRG) neurons. (A) Whole-cell patch clamp recordings of action potential (AP) amplitude and firing frequency in DRG neurons. (B) Calcium imaging of Ca^2+^ peak amplitude and event frequency. Scale bars, 25 μm. (C) Immunofluorescence staining of Growth-associated protein 43 (GAP43) expression. Scale bars, 25 μm. (D) Immunofluorescence staining of neurofilament 200 (NF200) expression. Scale bars, 25 μm. (E) Quantification of average axonal length and branching number based on axonal tracing. All experiments were repeated at least 3 times. ns, not significant; **P < 0.01; ***P < 0.001; ****P < 0.0001.

Whole-cell patch-clamp recordings revealed that LPS significantly reduced the amplitude and firing frequency of DRG neuron action potentials, while free IL-2 and MOF@PI only partially restored these parameters by about 0.18-fold (*P* < 0.05). By contrast, the LPS + MOF@PI-IL2 group showed a clear improvement in action potential characteristics by about 0.3-fold (*P* < 0.05). Notably, the LPS + MOF@PI empty-carrier group showed no significant improvement relative to the LPS model group (*P* > 0.05), indicating that the carrier alone does not confer neuroprotection (Fig. 5A).

Ca^2+^ imaging further supported this trend: LPS injury markedly suppressed both the peak amplitude and event frequency of Ca^2+^ transients by about 0.7-fold (*P* < 0.05), whereas MOF@PI-IL2 significantly restored Ca^2+^ activity and increased peak amplitude by about 1.2-fold (*P* < 0.05). In addition, the LPS + MOF@PI empty-carrier group showed no statistical difference from the LPS model group (*P* > 0.05), with no evidence of calcium signal recovery (Fig. 5B).

At the structural level, immunofluorescence staining showed that LPS markedly reduced GAP43 and NF200 expression, whereas free IL-2 provided limited improvement by about 0.9-fold (*P* < 0.05). In contrast, LPS + MOF@PI-IL2 coculture markedly enhanced signals for these regeneration- and maturation-associated nerve fiber markers. GAP43 and NF200 expression both increased, whereas the LPS + MOF@PI empty-carrier group remained comparable to the LPS model group (*P* > 0.05), showing no up-regulation of neuroregeneration markers (Fig. 5C and D).

Axonal tracing analysis further demonstrated that MOF@PI-IL2 substantially increased the morphological plasticity of DRG neurons, as indicated by increases in average axon length and branch number of about 1.5-fold (*P* < 0.05). In contrast, the LPS + MOF@PI empty-carrier group showed no significant difference from the LPS model group (*P* > 0.05), confirming that axonal structural reconstruction depends on IL-2-mediated Treg cell activation rather than the carrier itself (Fig. 5E).

Patch-clamp electrophysiology, Ca^2+^ imaging, and axonal tracing analyses collectively demonstrated that MOF@PI-IL2 improves the electrophysiological properties and structural plasticity of injured DRG neurons.

### Foxp3 KD significantly attenuates the Treg-cell-activating and neuroprotective effects of MOF@PI-IL2

To determine whether the neuroprotective effects of MOF@PI-IL2 depend on the Treg cell master regulator Foxp3, we performed rescue experiments using siFoxp3. The impact of Foxp3 depletion was evaluated in terms of Treg cell activation, cytokine secretion, neuronal electrophysiological recovery, calcium signaling, and expression of neuronal regeneration markers by comparing the MOF@PI-IL2 and MOF@PI-IL2 + siFoxp3 groups.

WB analysis first confirmed the efficiency of Foxp3 knockdown. Treatment with siFoxp3 significantly reduced Foxp3 protein levels and concurrently decreased STAT5 phosphorylation by about 0.3-fold (*P* < 0.05) (Fig. S2A).

We then assessed changes in the Treg cell immunosuppressive cytokine network. ELISA results showed that Foxp3 KD markedly decreased the secretion of IL-10 and TGF-β by Treg cells by about 0.5-fold (*P* < 0.05) (Fig. S2B), indicating that Foxp3 is a central hub for the anti-inflammatory efficacy induced by MOF@PI-IL2.

In the neuron-Treg cell coculture system, Foxp3 KD substantially impaired the neuroregenerative capacity of MOF@PI-IL2. Patch-clamp analysis showed that action potential amplitude and firing frequency recovery were insufficient, decreasing by about 0.35-fold relative to the nonknockdown group (*P* < 0.05) (Fig. S2C). Similarly, Ca^2+^ imaging showed that both calcium-peak amplitude and event frequency were markedly reduced by about 0.4-fold (*P* < 0.05), indicating substantial impairment of electrophysiological recovery (Fig. S2D).

Finally, immunofluorescence analysis showed only limited recovery of GAP43 and NF200 expression (Fig. S2E). Quantitative fluorescence analysis demonstrated that both GAP43 and NF200 signals decreased by about 0.7-fold (*P* < 0.05), accompanied by obvious insufficiency in axonal growth and branch reconstruction.

siRNA interference experiments demonstrated that Foxp3 KD weakens the Treg-cell-activating and neuroprotective effects of MOF@PI-IL2, indicating that its therapeutic efficacy depends on a Treg-cell-centric pathway.

### MOF@PI-IL2 significantly improves pain abnormalities and behavioral dysfunction in DPN mice

**Fig. 6.**
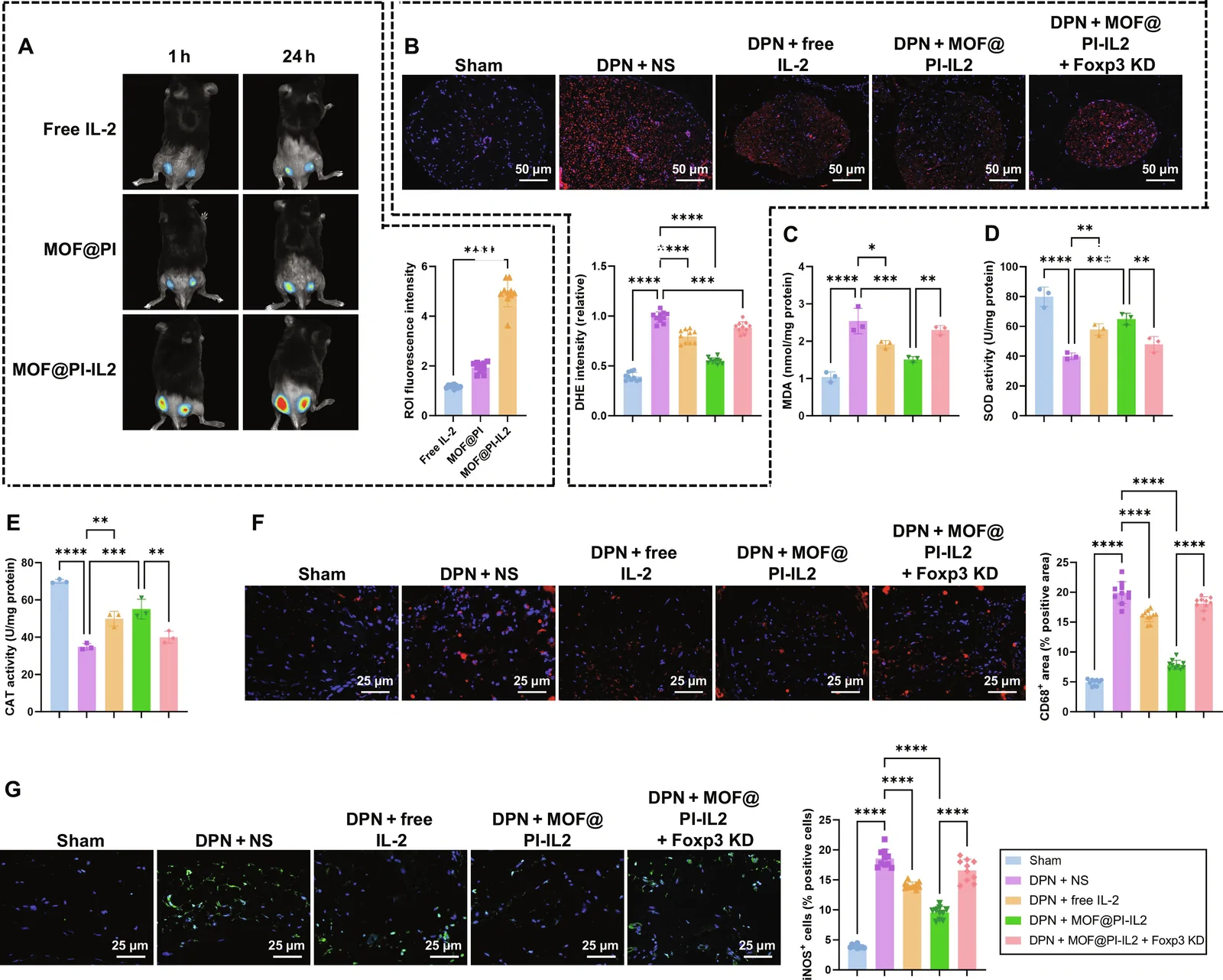
Preferential accumulation of polyimide-coated zeolitic imidazolate framework-8 core–shell nanovesicles loaded with interleukin-2 (IL-2) (MOF@PI-IL2) at sciatic nerve inflammatory sites in diabetic peripheral neuropathy (DPN) mice and alleviation of neuroinflammation. (A) In vivo imaging system (IVIS) of cyanine 5.5 (Cy5.5)-labeled free IL-2, MOF@PI, and MOF@PI-IL2 to assess in vivo tissue distribution in the sciatic nerves of DPN mice. (B) Dihydroethidium (DHE) fluorescence staining to evaluate oxidative stress. Scale bars, 50 μm. (C) Quantification of malondialdehyde (MDA) content. (D) Measurement of superoxide dismutase (SOD) activity. (E) Measurement of catalase (CAT) activity. (F) Immunofluorescence staining of CD68 to assess macrophage infiltration. Scale bars, 25 μm. (G) Immunofluorescence staining of inducible nitric oxide synthase (iNOS) to evaluate inflammatory molecule expression. Scale bar, 25 μm. *N* = 10. *P < 0.05; **P < 0.01; ***P < 0.001; ****P < 0.0001. ROI, region of interest.

First, in the hot plate test, DPN + NS mice displayed pronounced thermal hyperalgesia, with significantly shortened latency (about 0.4-fold, *P* < 0.05). The free IL-2 group showed only slight prolongation, whereas MOF@PI-IL2 significantly restored the thermal pain threshold by about 0.9-fold (*P* < 0.05) (Fig. 6A). Under Foxp3 KD conditions, this improvement was markedly weakened, with latency falling back to a level close to that of free IL-2, suggesting that recovery from thermal hyperalgesia depends on the Treg cell–Foxp3 pathway.

In the Von Frey test, DPN + NS mice showed a significant reduction in mechanical pain threshold, indicating mechanical hyperalgesia. free IL-2 yielded limited improvement, whereas MOF@PI-IL2 increased the mechanical threshold by about 1.3-fold (*P* < 0.05) (Fig. 6B). Improvement was insufficient in the Foxp3 KD group, further supporting a Treg-cell-dependent mode of action (*n* = 8).

Sensorimotor integration was then assessed using the toe spread/pinch reflex. DPN + NS mice responded sluggishly to stimulation, with significantly reduced toe-spread angle and reflex scores by about 0.7-fold (*P* < 0.05), whereas free IL-2 exerted only mild effects. MOF@PI-IL2 significantly improved toe spreading and reaction speed (*P* < 0.05), but this improvement was greatly reduced after Foxp3 KD (Fig. 6C), demonstrating that the material effectively restores peripheral sensory input and motor control.

Anxiety-like behavior was further assessed using the open field test. DPN + NS mice exhibited marked thigmotaxis, center-zone avoidance, and disorganized trajectories; free IL-2 yielded slight improvement, whereas MOF@PI-IL2 significantly increased time spent in the center by about 3.4-fold (*P* < 0.05), with more even trajectories and more stable locomotor activity (Fig. 6D). Behavioral improvement was markedly reduced in the Foxp3 KD group, supporting an association between anxiety relief and immunoregulation.

Finally, spatial working memory was evaluated using the Y-maze test. DPN + NS mice exhibited a significant decrease in spontaneous alternation rate by about 0.2-fold (*P* < 0.05). Free IL-2 produced limited recovery, whereas MOF@PI-IL2 significantly elevated the alternation rate by about 0.3-fold (*P* < 0.05) (Fig. 6E), approaching the sham level. Improvement remained insufficient in the Foxp3 KD group, indicating that cognitive recovery also depends on the Treg cell–Foxp3 axis.

Overall, hot plate, Von Frey, reflex, open-field, and Y-maze behavioral assessments demonstrated that MOF@PI-IL2 ameliorates multidimensional neurological dysfunction in DPN mice, whereas Foxp3 KD attenuates these behavioral benefits.

### MOF@PI-IL2 achieves preferential accumulation at sciatic nerve inflammatory sites in DPN mice and effectively alleviates local neuroinflammation

To determine whether MOF@PI-IL2 could achieve in vivo targeting and accumulation at peripheral sites of neuroinflammation and whether it effectively modulates focal neuroinflammatory responses associated with DPN, we conducted a series of experiments in a DPN mouse model. These included in vivo biodistribution imaging, tissue-level assessment of oxidative stress, and analysis of inflammatory cell infiltration. This section aimed to systematically evaluate the anti-inflammatory mechanism of MOF@PI-IL2 from 4 aspects: tissue localization, oxidative stress, immune cell infiltration, and Treg-cell-dependent pathways.

**Fig. 7.**
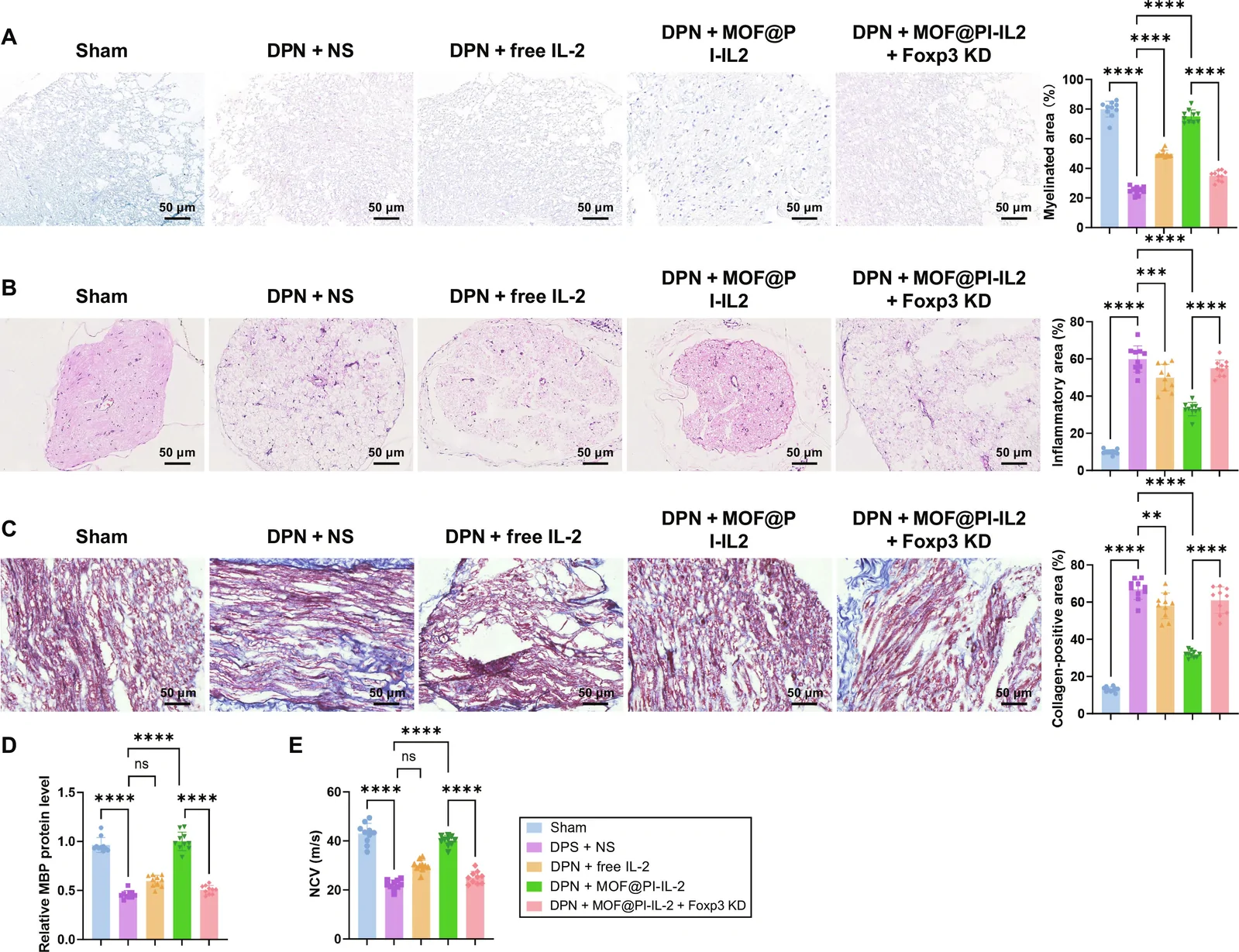
Polyimide-coated zeolitic imidazolate framework-8 core–shell nanovesicles loaded with interleukin-2 (MOF@PI-IL2) promotes myelin repair and improves nerve conduction function in diabetic peripheral neuropathy (DPN) mice. (A) Luxol Fast Blue (LFB) staining to assess myelin structure and myelinated area. Scale bars, 50 μm. (B) Hematoxylin and eosin (H&E) staining to evaluate sciatic nerve morphology and inflammatory cell infiltration. Scale bars, 50 μm. (C) Masson’s trichrome staining to assess collagen fiber deposition and the extent of fibrosis. Scale bars, 50 μm. (D) Western blot (WB) analysis of myelin basic protein (MBP) expression levels. (E) Nerve conduction velocity (NCV) testing to evaluate functional nerve conduction. *N* = 10. ns, not significant; **P < 0.01; ***P < 0.001; ****P < 0.0001.

First, Cy5.5 was used to label free IL-2, MOF@PI, and MOF@PI-IL2, and their tissue distribution was visualized using IVIS. As early as 1 h postinjection, MOF@PI-IL2 exhibited obvious fluorescence enrichment in the sciatic nerve region and maintained strong signals at 24 h (Fig. 7A). Regional fluorescence intensity was approximately 2.7-fold higher than that of free IL-2 and MOF@PI (*P* < 0.05), indicating excellent preferential accumulation and sustained retention in nerve tissue rather than absolute active targeting.

Subsequently, to comprehensively evaluate the regulatory effects of MOF@PI-IL2 on the local oxidative stress microenvironment in the sciatic nerves of DPN mice, we performed DHE fluorescence staining together with analyses of lipid peroxidation and antioxidant enzyme activities in sham, DPN + NS, DPN + free IL-2, DPN + MOF@PI-IL2, and DPN + MOF@PI-IL2 + Foxp3 KD mice. Compared with the sham group, the DPN+NS group exhibited pronounced oxidative stress, with markedly increased DHE fluorescence and MDA content (about 1.3-fold, *P* < 0.05), accompanied by significant decreases in SOD and CAT activities (about 0.5-fold, *P* < 0.05). Although free IL-2 produced partial improvement, MOF@PI-IL2 more effectively alleviated oxidative stress, whereas Foxp3 KD weakened this effect.

To further evaluate inflammatory cell infiltration, we examined CD68^+^ macrophage distribution in sciatic nerves. Compared with sham mice, the DPN + NS group displayed a large accumulation of CD68^+^ macrophages, increasing by about 2.7-fold (*P* < 0.05), whereas free IL-2 yielded only modest relief. MOF@PI-IL2 treatment significantly reduced CD68^+^ positivity by about 0.4-fold (*P* < 0.05) (Fig. 7F), but this effect was clearly weakened after Foxp3 KD, further emphasizing its dependence on immunoregulation.

At the molecular level of inflammation, iNOS immunofluorescence staining revealed marked enrichment of iNOS^+^ cells in the DPN + NS group. Free IL-2 produced only partial reduction, whereas MOF@PI-IL2 decreased the iNOS signal by about 0.4-fold (*P* < 0.05) (Fig. 7G). Foxp3 KD again substantially weakened this inhibitory trend, further supporting the idea that MOF@PI-IL2 suppresses inflammatory nitric oxide synthase activation through the Treg cell–Foxp3 pathway, thereby mitigating neuroinflammatory injury.

In vivo imaging, oxidative stress markers, and immunohistochemical analyses collectively demonstrated that MOF@PI-IL2 preferentially accumulates at sciatic nerve inflammatory sites and alleviates local neuroinflammation, whereas Foxp3 KD weakens this effect.

### MOF@PI-IL2 promotes myelin structure repair and improves nerve conduction function

To systematically evaluate whether MOF@PI-IL2 could repair peripheral nerve myelin damage and restore nerve conduction in DPN mice, we conducted experiments across 5 dimensions: myelin morphology, tissue inflammation, degree of fibrosis, myelin protein expression, and nerve electrophysiological function.

**Fig. 8.**
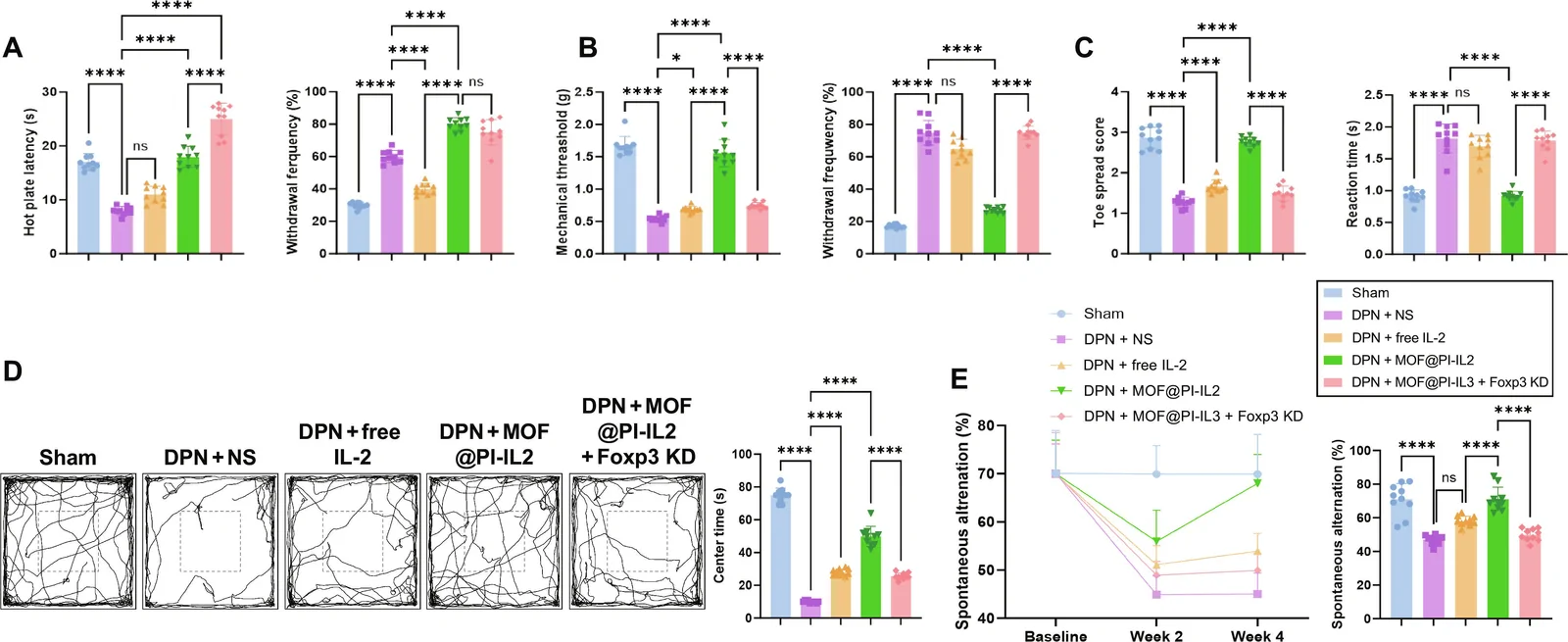
Polyimide-coated zeolitic imidazolate framework-8 core–shell nanovesicles loaded with interleukin-2 (MOF@PI-IL2) significantly improves pain abnormalities and behavioral function in diabetic peripheral neuropathy (DPN) mice. (A) Hot plate test measuring thermal pain threshold latency. (B) Von Frey test assessing mechanical pain threshold. (C) Toe spread/pinch test evaluating sensorimotor reflexes and toe-spreading scores. (D) Open field test assessing locomotor trajectories, time spent in the center, and anxiety-like behavior. (E) Y-maze test measuring spontaneous alternation to assess spatial working memory. *N* = 10; ns, not significant; *P < 0.05; ****P < 0.0001.

In LFB staining, the DPN + NS group exhibited typical demyelination features, including thinning, fragmentation, and disorganized alignment of myelin. Free IL-2 induced only mild improvement, whereas MOF@PI-IL2 significantly restored myelin continuity, thickness, and fiber regularity by about 1.4-fold (*P* < 0.05) (Fig. 8A). The myelinated area increased relative to DPN + NS, but this improvement was almost completely lost after Foxp3 KD, with reappearance of myelin defects, indicating dependence on the Treg cell–Foxp3 pathway.

H&E staining further reflected inflammatory tissue changes. The DPN + NS group showed marked inflammatory cell infiltration and swelling of nerve fascicles. Free IL-2 had limited effects, whereas MOF@PI-IL2 significantly reduced inflammatory cell accumulation and restored structural integrity, decreasing the inflammatory area by about 0.4-fold (*P* < 0.05) (Fig. 8B). This anti-inflammatory effect was again markedly weakened by Foxp3 KD.

In fibrosis assessment, Masson staining demonstrated extensive collagen deposition in the DPN + NS group, whereas free IL-2 only partially suppressed fibrosis. By contrast, MOF@PI-IL2 markedly reduced collagen accumulation, lowering the collagen-positive area by about 0.5-fold (*P* < 0.05) (Fig. 8C). The antifibrotic effect disappeared in the Foxp3 KD group, further supporting its dependence on immunoregulation.

At the molecular level, WB analysis of MBP showed that DPN induction resulted in substantial myelin protein loss. Free IL-2 provided only limited recovery, whereas MOF@PI-IL2 significantly up-regulated MBP expression by about 1.1-fold (*P* < 0.05) (Fig. 8D). In the Foxp3 KD group, MBP levels returned to nearly those of DPN + NS, indicating that myelin protein reconstruction depends on intact Treg cell signaling.

Finally, NCV tests were conducted to assess functional recovery. NCV was markedly reduced in the DPN + NS group by about 0.4-fold (*P* < 0.05). Free IL-2 produced partial recovery, whereas MOF@PI-IL2 significantly improved NCV by about 0.6-fold (*P* < 0.05) (Fig. 8E). However, improvement was substantially attenuated in the Foxp3 KD group, further confirming that functional nerve recovery arises from Treg cell–Foxp3-dependent immunoregulation and myelin reconstruction.

Histological staining, WB, and neurophysiological analyses collectively demonstrated that MOF@PI-IL2 promotes myelin structure repair and improves nerve conduction function, while Foxp3 KD weakens this restorative effect.

### Multiomics analysis reveals Treg cell depletion and impairment of the IL-2/Foxp3-STAT5 pathway in DPN nerve tissue, with partial restoration following MOF@PI-IL2 intervention

To investigate disruption of Treg cell immune homeostasis and impairment of the IL-2/Foxp3-STAT5 pathway in peripheral nerve tissue from DPN mice, we performed RNA sequencing (RNA-seq) on sciatic nerves from the sham, DPN + NS, and DPN + MOF@PI-IL2 groups. Differential expression analysis, immune infiltration profiling, and functional enrichment were integrated to define the local immunoregulatory landscape.

Differential expression analysis revealed a transcriptional signature of Treg cell exhaustion in the DPN + NS group relative to sham. Key Treg-cell-associated immunosuppressive genes, including Foxp3, STAT5B, IL2RA (CD25), and CTLA4, were markedly down-regulated, whereas IFNG, IL17A, and multiple inflammatory cytokines were up-regulated (Fig. S3A and B). In the DPN + MOF@PI-IL2 group, these Treg cell marker genes rebounded and expression of proinflammatory genes such as IFNG and IL17A was substantially reduced (Fig. S3C and D), suggesting that MOF@PI-IL2 remodels the Treg-cell-related transcriptional program while suppressing inflammatory pathways.

Immune infiltration analysis using CIBERSORT further showed a marked decrease in Treg cell infiltration in the DPN + NS group relative to sham, accompanied by increased proportions of Th1 cells, Th17 cells, and M1 macrophages, consistent with a proinflammatory immune landscape (Figs. S3E and S4A and B). In the DPN + MOF@PI-IL2 group, Treg cell infiltration scores increased significantly, while the proportions of Th1 cells, Th17 cells, and M1 macrophages decreased substantially, indicating reversal from a proinflammatory state toward enhanced immunoregulation and attenuated inflammation (Figs. S3F and S4C and D).

DEGs between the sham and DPN + NS groups were predominantly enriched in biological processes associated with neural dysfunction, including neurotransmitter transport, membrane potential regulation, synaptic structure, and ion channel activity (Fig. S3G). Corresponding KEGG pathways included neuroactive ligand–receptor interaction, cyclic guanosine monophosphate (cGMP)–cGMP-dependent protein kinase signaling, and multiple neurotransmission-related pathways, such as glutamatergic, serotonergic, and gamma-aminobutyric acid (GABA)ergic synapses (Fig. S3H). By contrast, DEGs between DPN + NS and DPN + MOF@PI-IL2 were mainly enriched in T cell differentiation, immunoreceptor activity, cytokine binding, and synapse-related processes (Fig. S3I), with KEGG pathways concentrated in Th1/Th2 cell differentiation, Th17 cell differentiation, hematopoietic lineage, and infection-related immunoregulatory networks (Fig. S3J), indicating that MOF@PI-IL2 may promote neural repair by reshaping the immunoregulatory axis.

In summary, these findings indicate that sciatic nerve tissue in DPN mice exhibits pronounced Treg cell depletion and suppression of the IL-2/Foxp3–STAT5 axis. MOF@PI-IL2 treatment partially reversed these abnormalities, supporting restoration of local immune homeostasis and attenuation of neuroinflammatory signaling.

### Multiomics integration identifies key regulatory networks involved in MOF@PI-IL2-mediated Treg cell activation and neuroinflammation alleviation

To further elucidate the mechanisms by which MOF@PI-IL2-induced Treg cell reactivation reshapes the neuroinflammatory microenvironment at the systems level, we conducted liquid chromatography–tandem mass spectrometry-based metabolomic profiling of mouse serum from the sham, DPN + NS, and DPN + MOF@PI-IL2 groups, followed by integration with RNA-seq data and construction of an IL-2 response prediction model.

Correlation analysis between differentially expressed metabolites and genes revealed that metabolic dysregulation between the sham and DPN + NS groups was mainly concentrated in the tryptophan–kynurenine pathway and short-chain fatty acid (SCFA) pathways (Figs. S5A and B and S6A). SCFA levels were significantly positively correlated with Treg cell marker gene expression (Fig. S5C), whereas kynurenine levels were negatively correlated with Treg cell genes and positively correlated with proinflammatory genes (Fig. S5D and E), indicating a close association between metabolic abnormality and Treg cell depletion. In the DPN + MOF@PI-IL2 group, SCFA levels increased relative to DPN + NS and tryptophan–kynurenine metabolic imbalance was partly corrected, accompanied by up-regulation of Treg cell marker genes and down-regulation of inflammation-related genes (Figs. S5F and G and S6B).

Building on these findings, we performed WGCNA to identify key coexpression modules driving Treg cell functional alterations. In the comparison between sham and DPN + NS, Foxp3, IL2RA, and other core Treg cell genes clustered in the green module, which was positively correlated with Treg cell infiltration and negatively correlated with M1 macrophage, Th1 cell, and Th17 cell infiltration (Figs. S5K and L and S6C to E). This module pattern reflects the typical DPN-associated state of overall down-regulation of Treg-cell-related gene sets and concomitant elevation of proinflammatory immune cells.

In contrast, WGCNA of the DPN + NS and DPN + MOF@PI-IL2 groups indicated that Foxp3, IL2RA, and other Treg cell core genes were enriched in the turquoise module. This module was likewise positively correlated with Treg cell infiltration and negatively correlated with M1 macrophage, Th1 cell, and Th17 cell infiltration (Figs. S5M and N and S6F to H). Notably, the overall direction of correlation was maintained, but its strength increased, suggesting that MOF@PI-IL2 enhances coordinated expression within Treg-cell-function-related modules while weakening gene expression connections associated with proinflammatory immune cells.

Integration of metabolomic and RNA-seq data revealed that Treg cell depletion in DPN is jointly driven by a triad of mechanisms: metabolic dysregulation, disruption of the Foxp3 coexpression module, and impaired IL-2 responsiveness. MOF@PI-IL2 intervention simultaneously corrected key metabolic pathways, including the tryptophan–kynurenine and SCFA pathways, restored the Foxp3-related coexpression module, and thereby facilitated Treg cell reactivation and alleviated neuroinflammation.

## Discussion

The role of Treg cells in maintaining immune homeostasis and modulating chronic inflammation has attracted broad attention in recent years, whereas their involvement in DPN remains insufficiently explored [29,30]. DPN has long been regarded primarily as a neuropathological complication driven by metabolic disturbance and oxidative stress, and most previous studies have focused on neuronal injury, repair, and symptomatic control [31]. However, accumulating evidence suggests that immune imbalance also contributes substantially to DPN progression. In this context, our findings support the view that the IL-2–Treg cell axis may represent an important therapeutic entry point in DPN.

Unlike previous studies that used direct IL-2 administration to activate Treg cells [32,33], the present study developed a MOF@PI-IL2 delivery system designed to improve IL-2 stability while reducing nonspecific immune activation. Although low-dose IL-2 has shown efficacy in several autoimmune diseases [34–36], its application in DPN remains limited by short half-life, unstable efficacy, and an insufficiently defined range of action. In our system, the acidic-microenvironment-responsive properties of the nanocarrier enabled preferential IL-2 release at inflammatory sites and favored uptake by Treg cells, thereby providing a more controlled mode of immune intervention.

From the perspective of carrier design, this study combined a MOF core with a PI-based interfacial condensation strategy to construct a stable core–shell nanostructure. Compared with conventional nanocarriers such as liposomes and poly(lactic-*co*-glycolic acid) [37,38], MOFs provide high surface area and structural tunability, which are advantageous for drug encapsulation and stimulus-responsive release. The PI shell further enhanced vesicle stability and conferred mild acid responsiveness, allowing the nanoplatform to remain stable under physiological conditions while promoting drug release in an inflammatory microenvironment. This integration of materials engineering with immunoregulation represents a key technical feature of the present work.

At the mechanistic level, the study demonstrated that MOF@PI-IL2 restores Treg cell function by activating the IL-2/STAT5/Foxp3 signaling pathway and reestablishes an anti-inflammatory factor network centered on IL-10 and TGF-β. The system effectively suppressed abnormal activation and cytotoxic secretion of CD8^+^ T cells and improved the inflammatory status of neural tissue. In the DRG neuron model, MOF@PI-IL2 also improved calcium signal stability and restored the expression of axonal markers, supporting a neuroprotective effect. Moreover, Foxp3 KD markedly weakened these protective effects, further indicating that the therapeutic activity of MOF@PI-IL2 depends largely on a Treg-cell-centered signaling axis.

At both the cellular and tissue levels, the present study showed that restoration of Treg cell function was associated with broad protective effects on the nervous system. In coculture systems, enhanced Treg cell activity suppressed CD8^+^ T cell cytotoxicity while preserving neuronal axonal extension and electrophysiological function. In animal experiments, MOF@PI-IL2 accumulated in the sciatic nerve, improved myelin structure, increased NCV, and ameliorated behavioral deficits. These findings suggest that Treg cell activation may not only correct immune imbalance but also create a permissive microenvironment for neural repair through intercellular immune–neural interactions.

It is worth emphasizing that this study integrated RNA-seq and metabolomics and obtained indicative evidence for a 3-dimensional “metabolism–immunity–neural” linkage. In DPN mice, reduced SCFAs and dysregulated tryptophan–kynurenine metabolism were observed, whereas MOF@PI-IL2 partly corrected these abnormalities and enhanced coexpression modules involving key genes such as Foxp3 and IL2RA. These findings suggest that metabolic disturbances can influence Treg cell fate and function, while immunoregulation may, in turn, improve metabolic status. This observation provides a new perspective for understanding the systems biology of DPN and immunometabolic intervention in chronic disease.

A study determined an effective low-dose regimen of 5,000 U/d via continuous intraperitoneal injection for 3 d in a rat chronic constriction injury model through dose screening [39]. This regimen differs in administration route and frequency from the MOF@PI-IL2 tail vein injection regimen of 2 μg per mouse per injection of IL-2 loading concentration used in this study, which was referenced from previous reports [40]. Considering that free IL-2 administered intraperitoneally is absorbed relatively slowly, this study ultimately selected tail vein injection, which can achieve rapid distribution. However, since intraperitoneal injection has the advantage of maintaining low-level exposure for a longer period, future studies will systematically measure the serum and tissue IL-2 concentration–time curves of different administration regimens to optimize the best therapeutic dose and administration frequency of MOF@PI-IL2 from the perspectives of pharmacokinetics and pharmacodynamics to clarify the potential advantages of the nanodelivery system over traditional low-dose regimens.

Compared with existing studies, the present work offers several innovations. This study proposes a Treg-cell-targeted nanoimmunotherapeutic strategy for DPN. At the technical level, it combines MOF and PI to achieve coordinated optimization of carrier stability and responsive release. At the mechanistic level, it links activation of the IL-2/STAT5/Foxp3 pathway with downstream immune restoration and neuroprotective effects. In addition, the study integrates behavioral, histological, and multiomics evidence, thereby providing a relatively comprehensive framework for evaluating therapeutic efficacy.

Several limitations should nevertheless be acknowledged. First, long-term safety and pharmacokinetic properties of the nanoplatform were not fully addressed in the present study. Second, IL-2 dose control and release kinetics may still require further optimization. Third, validation in large-animal models and additional preclinical toxicology studies will be necessary before translation can be considered. These issues should be addressed in future work to further define the therapeutic potential of this platform.

In conclusion, the MOF@PI-IL2 delivery system developed in this study enables inflammation-microenvironment-responsive IL-2 release and promotes restoration of Treg cell function, thereby alleviating neuroinflammation and functional impairment in DPN. This strategy therefore provides a translatable inflammation-microenvironment-responsive therapeutic platform for chronic neuroinflammatory disease.

## Ethical Approval

All animal experiments were approved by the Animal Ethics Committee of Xiangya Hospital (approval no. CSU-2022-0429).

## Data Availability

All data generated or analyzed during this study are included in this article and/or its Supplementary Materials. Further inquiries can be directed to the corresponding author.

## References

[1] Ko F-S, Wu T-H, Su G-Y, Lin Y-H, Juan C-C, Hwu C-M. Blood cell count-based inflammatory markers exhibit superior association with diabetic peripheral neuropathy compared to protein-based markers. J Inflamm Res. 2025;18:10609–10617. 40792223 10.2147/JIR.S524220 PMC12338494

[2] Gupta A, Gupta T, Singh TG, Singh R. Involvement of cellular and enzymatic aspects in the complexity of diabetic neuropathy. Tissue Barriers. 2025;14(2): Article 2581880. 41170890 10.1080/21688370.2025.2581880 PMC13228964

[3] Chong ZZ, Souayah N. Neuroinflammation in diabetic peripheral neuropathy and therapeutic implications. Rev Neurosci. 2025;36(7):749–762. 40228523 10.1515/revneuro-2025-0031

[4] Khanna S, Kumar S, Sharma P, Daksh R, Nandakumar K, Shenoy RR. Flavonoids regulating NLRP3 inflammasome: A promising approach in alleviating diabetic peripheral neuropathy. Inflammopharmacology. 2025;33(5):2231–2262. 40205269 10.1007/s10787-025-01729-7 PMC12176997

[5] Nica A-E, Andreea Parliteanu O, Claudia S, Dobjanschi C, Rusu E, Radulian G. Treatment options for diabetic neuropathy: Insights from the Romanian neuropathy guidelines. In: Carmignano SM, editor. *Peripheral neuropathy—Causes, symptoms, and treatment* options. London (UK): IntechOpen; 2025. p. 43–68.

[6] Khan S, Ahmad S, Khan M, Lohani M, Khan MS, Mohd H. Diabetic peripheral neuropathy: Navigating controversies and pioneering advances. Adv Life Sci. 2025;12(1):1–12.

[7] González H, Pacheco R. T-cell-mediated regulation of neuroinflammation involved in neurodegenerative diseases. J Neuroinflammation. 2014;11(1): Article 201. 25441979 10.1186/s12974-014-0201-8 PMC4258012

[8] Benamar M, Contini P, Schmitz-Abe K, Lanzetta O, Getachew F, Bachelin C, Leyva Castillo JM, Wang M, Oktelik FB, Perrot O, et al. Notch3 destabilizes regulatory T cells to drive autoimmune neuroinflammation in multiple sclerosis. Immunity. 2025;58(11):2753–2768.e6. 41043415 10.1016/j.immuni.2025.09.007 PMC12671433

[9] Kleinewietfeld M, Hafler DA. Regulatory T cells in autoimmune neuroinflammation. Immunol Rev. 2014;259(1):231–244. 24712469 10.1111/imr.12169 PMC3990868

[10] Schroeter CB, Huntemann N, Bock S, Nelke C, Kremer D, Pfeffer K, Meuth SG, Ruck T. Crosstalk of microorganisms and immune responses in autoimmune neuroinflammation: A focus on regulatory T cells. Front Immunol. 2021;12: Article 747143. 34691057 10.3389/fimmu.2021.747143 PMC8529161

[11] Olson KE, Mosley RL, Gendelman HE. The potential for treg-enhancing therapies in nervous system pathologies. Clin Exp Immunol. 2022;211(2):108–121. 10.1093/cei/uxac084 PMC10019130 36041453

[12] Faridar A, Thome AD, Zhao W, Thonhoff JR, Beers DR, Pascual B, Masdeu JC, Appel SH. Restoring regulatory T-cell dysfunction in Alzheimer’s disease through ex vivo expansion. Brain Commun. 2020;2(2): Article fcaa112. 32954348 10.1093/braincomms/fcaa112 PMC7472911

[13] Llorián-Salvador M, Pérez-Martínez D, Tang M, Duarri A, García-Ramirez M, Deàs-Just A, Álvarez-Guaita A, Ramos-Pérez L, Bogdanov P, Gomez-Sanchez JA, et al. Regulatory T cell expansion prevents retinal degeneration in type 2 diabetes. J Neuroinflammation. 2024;21(1): Article 328. 39716335 10.1186/s12974-024-03323-0 PMC11668053

[14] Huang Q, Zhu J. Regulatory T cell-based therapy in type 1 diabetes: Latest breakthroughs and evidence. Int Immunopharmacol. 2024;140: Article 112724. 39098233 10.1016/j.intimp.2024.112724

[15] Yue W, Sun N, Zhang J, Zhang W, Wu Y, Qu X, Zong J, Xu G. Alleviated diabetic osteoporosis and peripheral neuropathic pain by Rehmannia glutinosa Libosch polysaccharide via increasing regulatory T cells. Int J Biol Macromol. 2024;277: Article 134241. 39084449 10.1016/j.ijbiomac.2024.134241

[16] Raeber ME, Caspar DP, Zurbuchen Y, Guo N, Schmid J, Michler J, Martin AC, Steiner UC, Moor AE, Koning F, et al. Interleukin-2 immunotherapy reveals human regulatory T cell subsets with distinct functional and tissue-homing characteristics. Immunity. 2024;57(9):2232–2250.e10. 39137779 10.1016/j.immuni.2024.07.016

[17] Sun H, Lee HS, Kim SH, de Lima MF, Gingras AR, Du Q, McLaughlin W, Ablack J, Lopez-Ramirez MA, Lagarrigue F, et al. IL-2 can signal via chemokine receptors to promote regulatory T cells’ suppressive function. Cell Rep. 2023;42(8): Article 112996. 37598341 10.1016/j.celrep.2023.112996 PMC10564087

[18] Zhao TX, Sriranjan RS, Tuong ZK, Lu Y, Sage AP, Nus M, Hubsch A, Kaloyirou F, Vamvaka E, Helmy J, et al. Regulatory T-cell response to low-dose interleukin-2 in ischemic heart disease. NEJM Evid. 2022;1(1): Article EVID2100009. 10.1056/EVIDoa2100009 38319239

[19] Zhou Y, Quan G, Liu Y, Shi N, Wu Y, Zhang R, Gao X, Luo L. The application of interleukin-2 family cytokines in tumor immunotherapy research. Front Immunol. 2023;14: Article 1090311. 36936961 10.3389/fimmu.2023.1090311 PMC10018032

[20] Wang S, McGuirk CM, d’Aquino A, Mason JA, Mirkin CA. Metal–organic framework nanoparticles. Adv Mater. 2018;30(37): Article 1800202. 10.1002/adma.201800202 29862586

[21] Cao J, Li X, Tian H. Metal-organic framework (MOF)-based drug delivery. Curr Med Chem. 2020;27(35):5949–5969. 31215374 10.2174/0929867326666190618152518

[22] Shortall K, Otero F, Bendl S, Soulimane T, Magner E. Enzyme immobilization on metal organic frameworks: The effect of buffer on the stability of the support. Langmuir. 2022;38(44):13382–13391. 36286410 10.1021/acs.langmuir.2c01630 PMC9648341

[23] Carrillo-Carrión C, Comaills V, Visiga AM, Gauthier BR, Khiar N. Enzyme-responsive Zr-based metal–organic frameworks for controlled drug delivery: Taking advantage of clickable PEG-phosphate ligands. ACS Appl Mater Interfaces. 2023;15(23):27600–27611. 37249914 10.1021/acsami.3c03230 PMC10273175

[24] Fatima SF, Sabouni R, Husseini G, Paul V, Gomaa H, Radha R. Microwave-responsive metal-organic frameworks (MOFs) for enhanced in vitro controlled release of doxorubicin. Nanomaterials. 2024;14(13):1081. 38998686 10.3390/nano14131081 PMC11243425

[25] Li Z, Huang J, Wu J. pH-sensitive nanogels for drug delivery in cancer therapy. Biomater Sci. 2021;9(3):574–589. 33306076 10.1039/d0bm01729a

[26] Zandieh MA, Farahani MH, Daryab M, Motahari A, Gholami S, Salmani F, Karimi F, Samaei SS, Rezaee A, Rahmanian P, et al. Stimuli-responsive (nano)architectures for phytochemical delivery in cancer therapy. Biomed Pharmacother. 2023;166: Article 115283. 37567073 10.1016/j.biopha.2023.115283

[27] Wang L, Lin X, Sheng Y, Zhu H, Li Z, Su Z, Yu R, Zhang S. Synthesis of a crystalline zeolitic imidazole framework-8 nano-coating on single environment-sensitive viral particles for enhanced immune responses. Nanoscale Adv. 2023;5(5):1433–1449. 36866262 10.1039/d2na00767c PMC9972853

[28] Wang Q, Sun Y, Li S, Zhang P, Yao Q. Synthesis and modification of ZIF-8 and its application in drug delivery and tumor therapy. RSC Adv. 2020;10(62):37600–37620. 35515141 10.1039/d0ra07950b PMC9057214

[29] Neuwirth T, Malzl D, Knapp K, Tsokkou P, Kleissl L, Gabriel A, Reininger B, Freystätter C, Marella N, Kutschat AP, et al. The polyamine-regulating enzyme SSAT1 impairs tissue regulatory T cell function in chronic cutaneous inflammation. Immunity. 2025;58(3):632–647.e12. 40023161 10.1016/j.immuni.2025.02.011

[30] Bittner S, Hehlgans T, Feuerer M. Engineered Treg cells as putative therapeutics against inflammatory diseases and beyond. Trends Immunol. 2023;44(6):468–483. 37100644 10.1016/j.it.2023.04.005

[31] Yang Y, Zhao B, Wang Y, Lan H, Liu X, Hu Y, Cao P. Diabetic neuropathy: Cutting-edge research and future directions. Signal Transduct Target Ther. 2025;10(1):132. 40274830 10.1038/s41392-025-02175-1 PMC12022100

[32] Harris F, Berdugo YA, Tree T. IL-2-based approaches to Treg enhancement. Clin Exp Immunol. 2022;211(2):149–163. 10.1093/cei/uxac105 PMC10019135 36399073

[33] Xu L, Song X, Su L, Zheng Y, Li R, Sun J. New therapeutic strategies based on IL-2 to modulate Treg cells for autoimmune diseases. Int Immunopharmacol. 2019;72:322–329. 31005777 10.1016/j.intimp.2019.03.064

[34] Zhang R, Zhao Y, Chen X, Zhuang Z, Li X, Shen E. Low-dose IL-2 therapy in autoimmune diseases: An update review. Int Rev Immunol. 2024;43(3):113–137. 37882232 10.1080/08830185.2023.2274574

[35] Tahvildari M, Dana R. Low-dose IL-2 therapy in transplantation, autoimmunity, and inflammatory diseases. J Immunol. 2019;203(11):2749–2755. 31740549 10.4049/jimmunol.1900733 PMC6986328

[36] Graßhoff H, Comdühr S, Monne LR, Müller A, Lamprecht P, Riemekasten G, Humrich JY. Low-dose IL-2 therapy in autoimmune and rheumatic diseases. Front Immunol. 2021;12: Article 648408. 33868284 10.3389/fimmu.2021.648408 PMC8047324

[37] Danhier F, Ansorena E, Silva JM, Coco R, Le Breton A, Préat V. PLGA-based nanoparticles: An overview of biomedical applications. J Control Release. 2012;161(2):505–522. 22353619 10.1016/j.jconrel.2012.01.043

[38] Fidan Y, Muçaj S, Timur SS, Gürsoy RN. Recent advances in liposome-based targeted cancer therapy. J Liposome Res. 2023;34(2):316–334. 37814217 10.1080/08982104.2023.2268710

[39] Zhao Y, Shen L, Huang Y. Low-dose IL-2 attenuates neuropathic pain via Treg expansion in rats. J Pain Res. 2025;18:5011–5022. 41035801 10.2147/JPR.S550012 PMC12482938

[40] Schultz KR, Badger CC, Dombi GW, Greenberg PD, Bernstein ID. Effect of interleukin-2 on biodistribution of monoclonal antibody in tumor and normal tissues in mice bearing SL-2 thymoma. JNCI J Natl Cancer Inst. 1992;84(2):109–113. 1735876 10.1093/jnci/84.2.109

