# Supplementary material for: Acid-Responsive Zeolitic Imidazolate Framework-8 Core–Shell Nanovesicles Loaded with Interleukin-2 Nanoplatforms Selectively Expand Regulatory T Cells to Reconstruct Immunometabolic Homeostasis in Diabetic Neuropathy
Source: Biomater Res. 2026 Sep 4;30:0407. doi: 10.34133/bmr.0407 (PMC13542432; doi:10.34133/bmr.0407)
Supplement: Supplementary file 1 [file bmr.0407.f1.zip › figure S1.pdf]

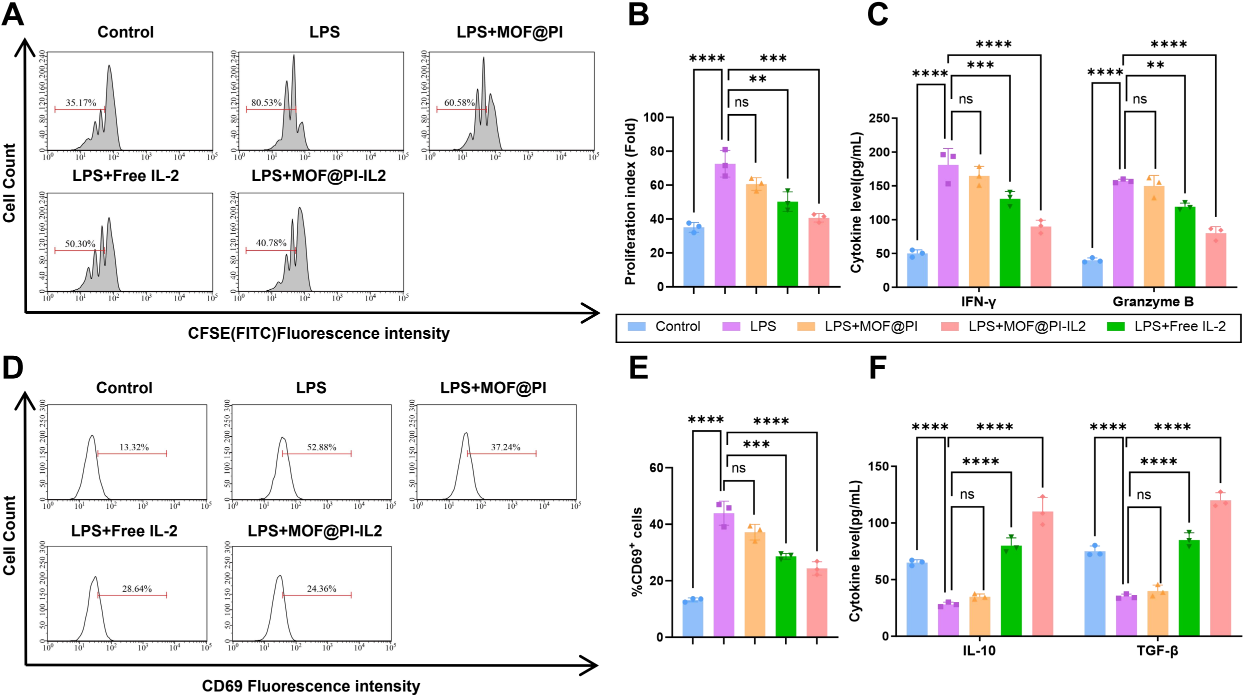

A
Control
LPS
LPS+MOF@PI
LPS+Free IL-2
LPS+MOF@PI-IL2
35.17%
80.53%
60.58%
50.30%
40.78%
Cell Count
CFSE(FITC)Fluorescence intensity
B
Proliferation index (Fold)
C
Cytokine level(pg/mL)
IFN-γ
Granzyme B
Control
LPS
LPS+MOF@PI
LPS+MOF@PI-IL2
LPS+Free IL-2
D
13.32%
52.88%
37.24%
28.64%
24.36%
CD69 Fluorescence intensity
E
%CD69+ cells
F
IL-10
TGF-β
