# Supplementary material for: Acid-Responsive Zeolitic Imidazolate Framework-8 Core–Shell Nanovesicles Loaded with Interleukin-2 Nanoplatforms Selectively Expand Regulatory T Cells to Reconstruct Immunometabolic Homeostasis in Diabetic Neuropathy
Source: Biomater Res. 2026 Sep 4;30:0407. doi: 10.34133/bmr.0407 (PMC13542432; doi:10.34133/bmr.0407)
Supplement: Supplementary file 1 [file bmr.0407.f1.zip › figure S2.pdf]

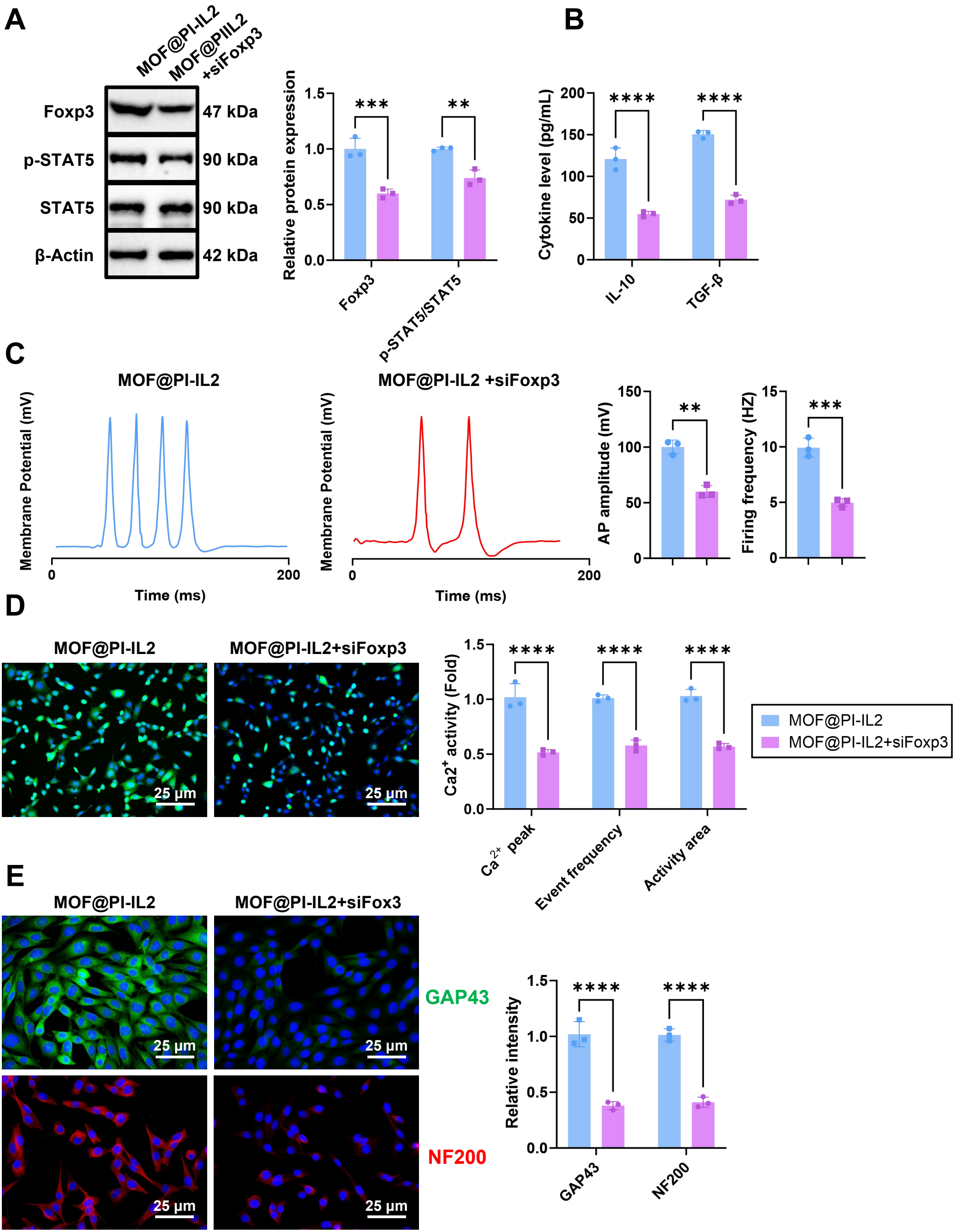

A
MOF@PI-IL2
MOF@PIIL2 +siFoxp3
Foxp3
p-STAT5
STAT5
β-Actin
47 kDa
90 kDa
90 kDa
42 kDa
Relative protein expression
***
**
Foxp3
p-STAT5/STAT5
B
Cytokine level (pg/mL)
****
****
IL-10
TGF-β
C
MOF@PI-IL2
MOF@PI-IL2 +siFoxp3
Membrane Potential (mV)
Time (ms)
0
200
AP amplitude (mV)
Firing frequency (HZ)
**
***
D
MOF@PI-IL2
MOF@PI-IL2+siFoxp3
25 μm
Ca2+ activity (Fold)
Ca2+ peak
Event frequency
Activity area
MOF@PI-IL2
MOF@PI-IL2+siFoxp3
E
MOF@PI-IL2
MOF@PI-IL2+siFox3
GAP43
NF200
Relative intensity
