# Supplementary material for: Acid-Responsive Zeolitic Imidazolate Framework-8 Core–Shell Nanovesicles Loaded with Interleukin-2 Nanoplatforms Selectively Expand Regulatory T Cells to Reconstruct Immunometabolic Homeostasis in Diabetic Neuropathy
Source: Biomater Res. 2026 Sep 4;30:0407. doi: 10.34133/bmr.0407 (PMC13542432; doi:10.34133/bmr.0407)
Supplement: Supplementary file 1 [file bmr.0407.f1.zip › figure S3.pdf]

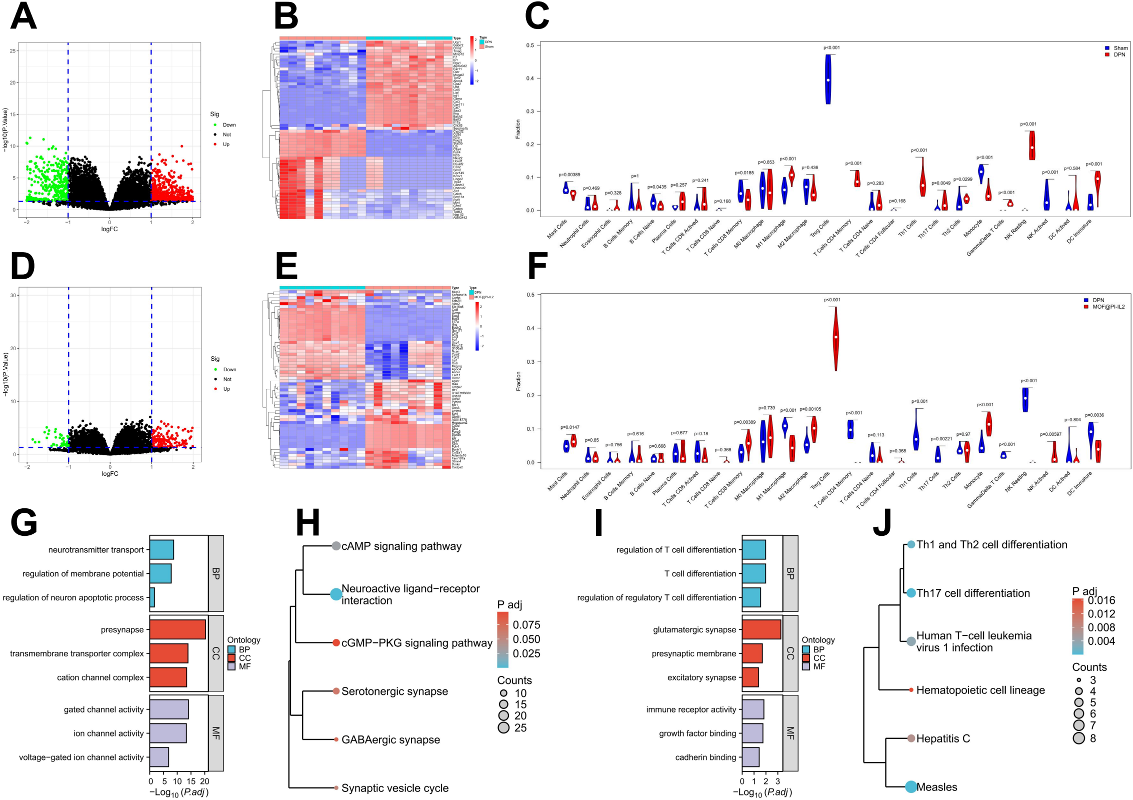

A
B
C
D
E
F
G
H
I
J
logFC
−log10(P.Value)
Sig
Down
Not
Up
Type
DPN
Sham
MOF@PI-IL2
Fraction
Mast Cells
Neutrophil Cells
Eosinophil Cells
B Cells Memory
B Cells Naive
Plasma Cells
T Cells CD8 Actived
T Cells CD8 Naive
T Cells CD8 Memory
M0 Macrophage
M1 Macrophage
M2 Macrophage
Treg Cells
T Cells CD4 Memory
T Cells CD4 Naive
T Cells CD4 Follicular
Th1 Cells
Th17 Cells
Th2 Cells
Monocyte
GammaDelta T Cells
NK Resting
NK Actived
DC Actived
DC Immature
neurotransmitter transport
regulation of membrane potential
regulation of neuron apoptotic process
presynapse
transmembrane transporter complex
cation channel complex
gated channel activity
ion channel activity
voltage−gated ion channel activity
BP
CC
MF
Ontology
−Log10 (P.adj)
cAMP signaling pathway
Neuroactive ligand−receptor interaction
cGMP−PKG signaling pathway
Serotonergic synapse
GABAergic synapse
Synaptic vesicle cycle
P adj
Counts
regulation of T cell differentiation
T cell differentiation
regulation of regulatory T cell differentiation
glutamatergic synapse
presynaptic membrane
excitatory synapse
immune receptor activity
growth factor binding
cadherin binding
Th1 and Th2 cell differentiation
Th17 cell differentiation
Human T−cell leukemia virus 1 infection
Hematopoietic cell lineage
Hepatitis C
Measles
