# Supplementary material for: Acid-Responsive Zeolitic Imidazolate Framework-8 Core–Shell Nanovesicles Loaded with Interleukin-2 Nanoplatforms Selectively Expand Regulatory T Cells to Reconstruct Immunometabolic Homeostasis in Diabetic Neuropathy
Source: Biomater Res. 2026 Sep 4;30:0407. doi: 10.34133/bmr.0407 (PMC13542432; doi:10.34133/bmr.0407)
Supplement: Supplementary file 1 [file bmr.0407.f1.zip › figure S4.pdf]

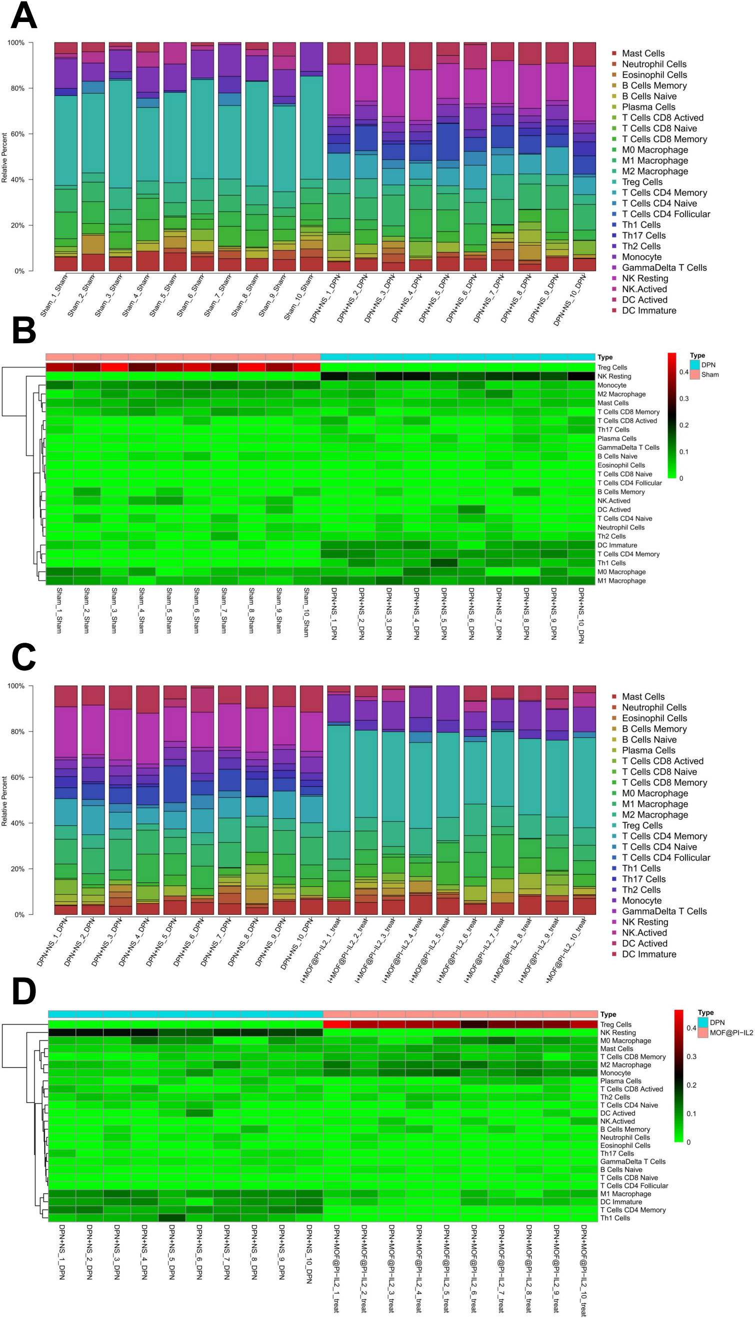
A
100%
80%
60%
40%
20%
0%
Relative Percent
Sham_1_Sham
Sham_2_Sham
Sham_3_Sham
Sham_4_Sham
Sham_5_Sham
Sham_6_Sham
Sham_7_Sham
Sham_8_Sham
Sham_9_Sham
Sham_10_Sham
DPN+NS_1_DPN
DPN+NS_2_DPN
DPN+NS_3_DPN
DPN+NS_4_DPN
DPN+NS_5_DPN
DPN+NS_6_DPN
DPN+NS_7_DPN
DPN+NS_8_DPN
DPN+NS_9_DPN
DPN+NS_10_DPN
Mast Cells
Neutrophil Cells
Eosinophil Cells
B Cells Memory
B Cells Naive
Plasma Cells
T Cells CD8 Actived
T Cells CD8 Naive
T Cells CD8 Memory
M0 Macrophage
M1 Macrophage
M2 Macrophage
Treg Cells
T Cells CD4 Memory
T Cells CD4 Naive
T Cells CD4 Follicular
Th1 Cells
Th17 Cells
Th2 Cells
Monocyte
GammaDelta T Cells
NK Resting
NK.Actived
DC Actived
DC Immature
B
Type
Treg Cells
NK Resting
Monocyte
M2 Macrophage
Mast Cells
T Cells CD8 Memory
T Cells CD8 Actived
Th17 Cells
Plasma Cells
GammaDelta T Cells
B Cells Naive
Eosinophil Cells
T Cells CD8 Naive
T Cells CD4 Follicular
B Cells Memory
NK.Actived
DC Actived
T Cells CD4 Naive
Neutrophil Cells
Th2 Cells
DC Immature
T Cells CD4 Memory
Th1 Cells
M0 Macrophage
M1 Macrophage
0.4
0.3
0.2
0.1
0
Type
DPN
Sham
Sham_1_Sham
Sham_2_Sham
Sham_3_Sham
Sham_4_Sham
Sham_5_Sham
Sham_6_Sham
Sham_7_Sham
Sham_8_Sham
Sham_9_Sham
Sham_10_Sham
DPN+NS_1_DPN
DPN+NS_2_DPN
DPN+NS_3_DPN
DPN+NS_4_DPN
DPN+NS_5_DPN
DPN+NS_6_DPN
DPN+NS_7_DPN
DPN+NS_8_DPN
DPN+NS_9_DPN
DPN+NS_10_DPN
C
100%
80%
60%
40%
20%
0%
Relative Percent
DPN+NS_1_DPN
DPN+NS_2_DPN
DPN+NS_3_DPN
DPN+NS_4_DPN
DPN+NS_5_DPN
DPN+NS_6_DPN
DPN+NS_7_DPN
DPN+NS_8_DPN
DPN+NS_9_DPN
DPN+NS_10_DPN
I+MOF@PI−IL2_1_treat
I+MOF@PI−IL2_2_treat
I+MOF@PI−IL2_3_treat
I+MOF@PI−IL2_4_treat
I+MOF@PI−IL2_5_treat
I+MOF@PI−IL2_6_treat
I+MOF@PI−IL2_7_treat
I+MOF@PI−IL2_8_treat
I+MOF@PI−IL2_9_treat
MOF@PI−IL2_10_treat
Mast Cells
Neutrophil Cells
Eosinophil Cells
B Cells Memory
B Cells Naive
Plasma Cells
T Cells CD8 Actived
T Cells CD8 Naive
T Cells CD8 Memory
M0 Macrophage
M1 Macrophage
M2 Macrophage
Treg Cells
T Cells CD4 Memory
T Cells CD4 Naive
T Cells CD4 Follicular
Th1 Cells
Th17 Cells
Th2 Cells
Monocyte
GammaDelta T Cells
NK Resting
NK.Actived
DC Actived
DC Immature
D
Type
Treg Cells
NK Resting
M0 Macrophage
Mast Cells
T Cells CD8 Memory
M2 Macrophage
Monocyte
Plasma Cells
T Cells CD8 Actived
Th2 Cells
T Cells CD4 Naive
DC Actived
NK.Actived
B Cells Memory
Neutrophil Cells
Eosinophil Cells
Th17 Cells
GammaDelta T Cells
B Cells Naive
T Cells CD8 Naive
T Cells CD4 Follicular
M1 Macrophage
DC Immature
T Cells CD4 Memory
Th1 Cells
0.4
0.3
0.2
0.1
0
Type
DPN
MOF@PI−IL2
DPN+NS_1_DPN
DPN+NS_2_DPN
DPN+NS_3_DPN
DPN+NS_4_DPN
DPN+NS_5_DPN
DPN+NS_6_DPN
DPN+NS_7_DPN
DPN+NS_8_DPN
DPN+NS_9_DPN
DPN+NS_10_DPN
DPN+MOF@PI−IL2_1_treat
DPN+MOF@PI−IL2_2_treat
DPN+MOF@PI−IL2_3_treat
DPN+MOF@PI−IL2_4_treat
DPN+MOF@PI−IL2_5_treat
DPN+MOF@PI−IL2_6_treat
DPN+MOF@PI−IL2_7_treat
DPN+MOF@PI−IL2_8_treat
DPN+MOF@PI−IL2_9_treat
DPN+MOF@PI−IL2_10_treat
