# Supplementary material for: Acid-Responsive Zeolitic Imidazolate Framework-8 Core–Shell Nanovesicles Loaded with Interleukin-2 Nanoplatforms Selectively Expand Regulatory T Cells to Reconstruct Immunometabolic Homeostasis in Diabetic Neuropathy
Source: Biomater Res. 2026 Sep 4;30:0407. doi: 10.34133/bmr.0407 (PMC13542432; doi:10.34133/bmr.0407)
Supplement: Supplementary file 1 [file bmr.0407.f1.zip › figure S5.pdf]

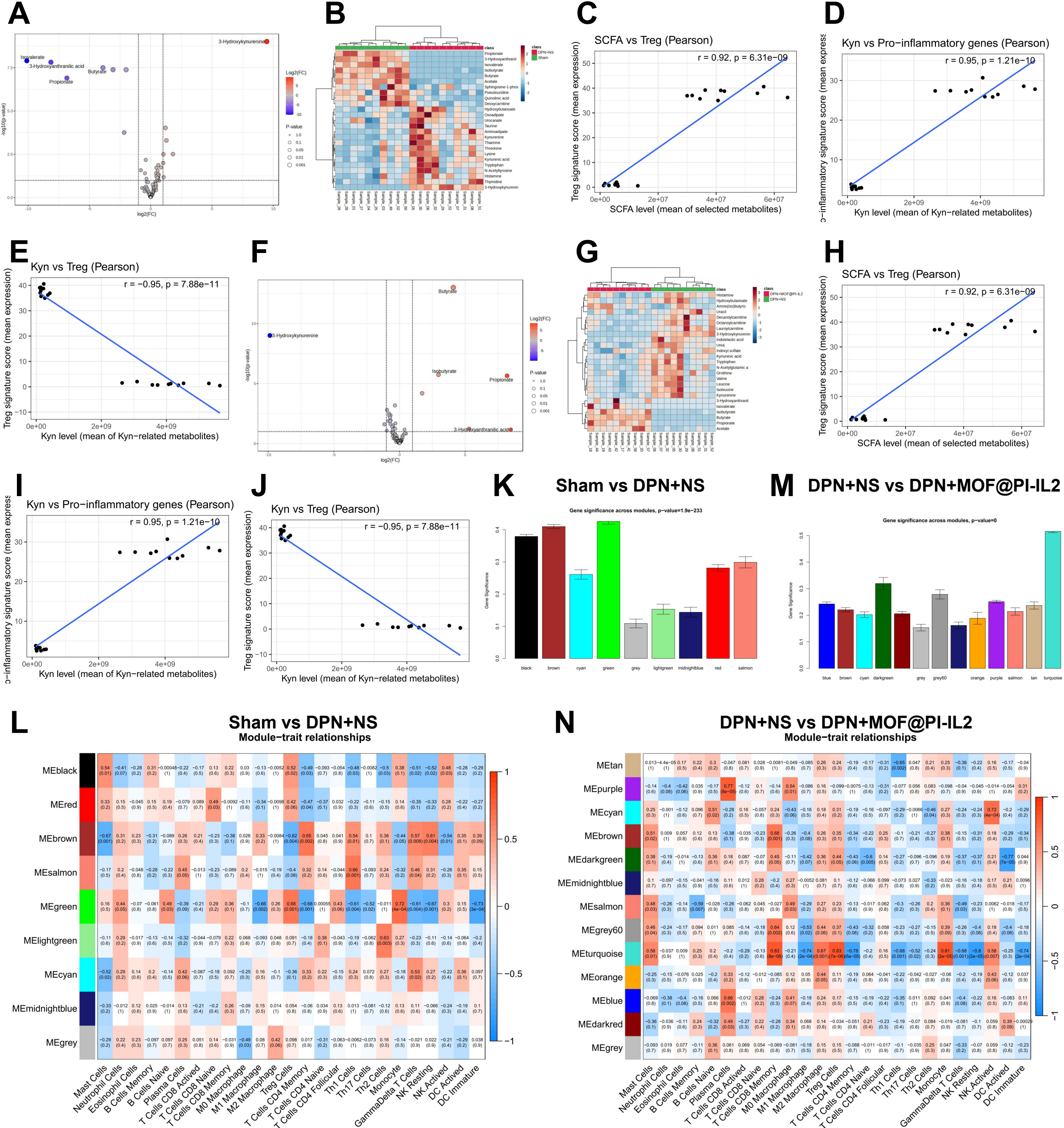
A
3-Hydroxykynurenine
Isovalerate
3-Hydroxyanthranilic acid
Butyrate
Propionate
-log10(p-value)
log2(FC)
Log2(FC)
P-value
B
class
DPN+NS
Sham
Propionate
3-Hydroxyanthranil
Isovalerate
Isobutyrate
Butyrate
Acetate
Sphingosine-1-phos
Pseudouridine
Quinolinic acid
Deoxycarnitine
Hydroxybutanoate
Oxoadipate
Urocanate
Taurine
Aminoadipate
Kynurenine
Thiamine
Threonine
Lysine
Kynurenic acid
Tryptophan
N-Acetyltyrosine
Histamine
Thymidine
3-Hydroxykynurenin
C
SCFA vs Treg (Pearson)
r = 0.92, p = 6.31e−09
Treg signature score (mean expression)
SCFA level (mean of selected metabolites)
D
Kyn vs Pro−inflammatory genes (Pearson)
r = 0.95, p = 1.21e−10
Kyn level (mean of Kyn−related metabolites)
E
Kyn vs Treg (Pearson)
r = −0.95, p = 7.88e−11
Treg signature score (mean expression)
Kyn level (mean of Kyn−related metabolites)
F
Butyrate
3-Hydroxykynurenine
Isobutyrate
Propionate
3-Hydroxyanthranilic acid
-log10(p-value)
log2(FC)
Log2(FC)
P-value
G
class
DPN+MOF@PI-IL2
DPN+NS
Histamine
Hydroxybutanoate
Amino(iso)butyric
Uracil
Decanoylcarnitine
Octanoylcarnitine
Lauroylcarnitine
3-Hydroxykynurenin
Indolelactic acid
Urea
Indoxyl sulfate
Kynurenic acid
Tryptophan
N-Acetylglutamic a
Ornithine
Valine
Leucine
Isoleucine
Kynurenine
3-Hydroxyanthranil
Isovalerate
Isobutyrate
Butyrate
Propionate
Acetate
H
SCFA vs Treg (Pearson)
r = 0.92, p = 6.31e−09
Treg signature score (mean expression)
SCFA level (mean of selected metabolites)
I
Kyn vs Pro−inflammatory genes (Pearson)
r = 0.95, p = 1.21e−10
Kyn level (mean of Kyn−related metabolites)
J
Kyn vs Treg (Pearson)
r = −0.95, p = 7.88e−11
Treg signature score (mean expression)
Kyn level (mean of Kyn−related metabolites)
K
Sham vs DPN+NS
Gene significance across modules, p−value=1.9e−233
Gene Significance
black
brown
cyan
green
grey
lightgreen
midnightblue
red
salmon
M
DPN+NS vs DPN+MOF@PI-IL2
Gene significance across modules, p−value=0
Gene Significance
blue
brown
cyan
darkgreen
grey
grey60
orange
purple
salmon
tan
turquoise
L
Sham vs DPN+NS
Module−trait relationships
MEblack
MEred
MEbrown
MEsalmon
MEgreen
MElightgreen
MEcyan
MEmidnightblue
MEgrey
N
DPN+NS vs DPN+MOF@PI-IL2
Module−trait relationships
MEtan
MEpurple
MEcyan
MEbrown
MEdarkgreen
MEmidnightblue
MEsalmon
MEgrey60
MEturquoise
MEorange
MEblue
MEdarkred
MEgrey
Mast Cells
Neutrophil Cells
Eosinophil Cells
B Cells Memory
B Cells Naive
Plasma Cells
T Cells CD8 Actived
T Cells CD8 Naive
T Cells CD8 Memory
M0 Macrophage
M1 Macrophage
M2 Macrophage
Treg Cells
T Cells CD4 Memory
T Cells CD4 Naive
T Cells CD4 Follicular
Th1 Cells
Th17 Cells
Th2 Cells
Monocyte
GammaDelta T Cells
NK Resting
NK Actived
DC Actived
DC Immature
