# Supplementary material for: Acid-Responsive Zeolitic Imidazolate Framework-8 Core–Shell Nanovesicles Loaded with Interleukin-2 Nanoplatforms Selectively Expand Regulatory T Cells to Reconstruct Immunometabolic Homeostasis in Diabetic Neuropathy
Source: Biomater Res. 2026 Sep 4;30:0407. doi: 10.34133/bmr.0407 (PMC13542432; doi:10.34133/bmr.0407)
Supplement: Supplementary file 1 [file bmr.0407.f1.zip › figure S6.pdf]

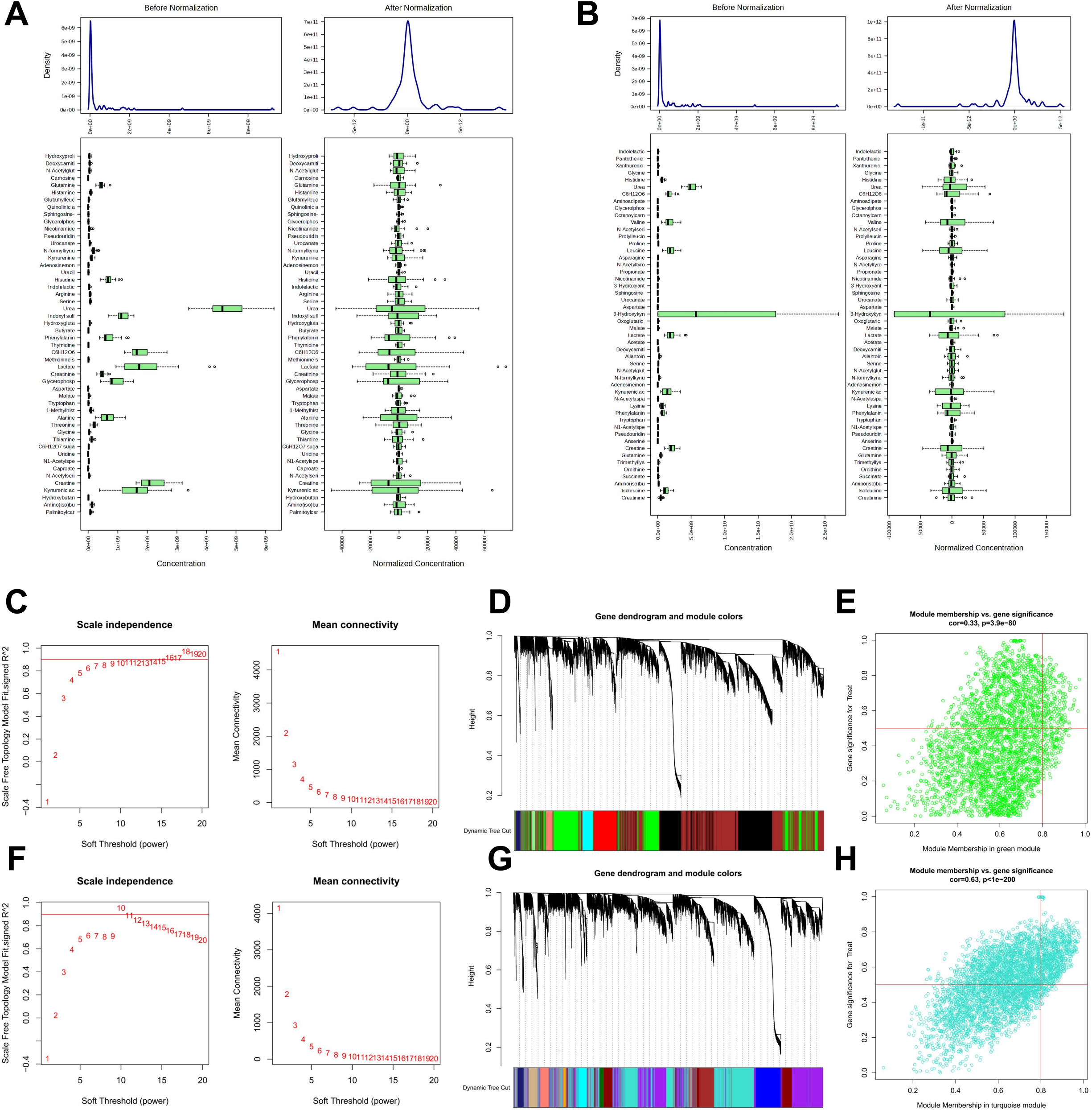
A
Before Normalization
After Normalization
Density
Hydroxyproli
Deoxycarniti
N-Acetylglut
Carnosine
Glutamine
Histamine
Glutamylleuc
Quinolinic a
Sphingosine-
Glycerolphos
Nicotinamide
Pseudouridin
Urocanate
N-formylkynu
Kynurenine
Adenosinemon
Uracil
Histidine
Indolelactic
Arginine
Serine
Urea
Indoxyl sulf
Hydroxygluta
Butyrate
Phenylalanin
Thymidine
C6H12O6
Methionine s
Lactate
Creatinine
Glycerophosp
Aspartate
Malate
Tryptophan
1-Methylhist
Alanine
Threonine
Glycine
Thiamine
C6H12O7 suga
Uridine
N1-Acetylspe
Caproate
N-Acetylseri
Creatine
Kynurenic ac
Hydroxybutan
Amino(iso)bu
Palmitoylcar
Concentration
Normalized Concentration
B
Before Normalization
After Normalization
Density
Indolelactic
Pantothenic
Xanthurenic
Glycine
Histidine
Urea
C6H12O6
Aminoadipate
Glycerolphos
Octanoylcarn
Valine
N-Acetylseri
Prolylleucin
Proline
Leucine
Asparagine
N-Acetyltyro
Propionate
Nicotinamide
3-Hydroxyant
Sphingosine
Urocanate
Aspartate
3-Hydroxykyn
Oxoglutaric
Malate
Lactate
Acetate
Deoxycarniti
Allantoin
Serine
N-Acetylglut
N-formylkynu
Adenosinemon
Kynurenic ac
N-Acetylaspa
Lysine
Phenylalanin
Tryptophan
N1-Acetylspe
Pseudouridin
Anserine
Creatine
Glutamine
Trimethyllys
Ornithine
Succinate
Amino(iso)bu
Isoleucine
Creatinine
Concentration
Normalized Concentration
C
Scale independence
Scale Free Topology Model Fit,signed R^2
Soft Threshold (power)
Mean connectivity
Mean Connectivity
Soft Threshold (power)
D
Gene dendrogram and module colors
Height
Dynamic Tree Cut
E
Module membership vs. gene significance
cor=0.33, p=3.9e−80
Gene significance for Treat
Module Membership in green module
F
Scale independence
Scale Free Topology Model Fit,signed R^2
Soft Threshold (power)
Mean connectivity
Mean Connectivity
Soft Threshold (power)
G
Gene dendrogram and module colors
Height
Dynamic Tree Cut
H
Module membership vs. gene significance
cor=0.63, p<1e−200
Gene significance for Treat
Module Membership in turquoise module
