# Supplementary material for: Acid-Responsive Zeolitic Imidazolate Framework-8 Core–Shell Nanovesicles Loaded with Interleukin-2 Nanoplatforms Selectively Expand Regulatory T Cells to Reconstruct Immunometabolic Homeostasis in Diabetic Neuropathy
Source: Biomater Res. 2026 Sep 4;30:0407. doi: 10.34133/bmr.0407 (PMC13542432; doi:10.34133/bmr.0407)
Supplement: Supplementary file 1 [file bmr.0407.f1.zip › sm caption.pdf]

Supplementary Figure Legends

Figure S1. MOF@PI-IL2 enhances Treg suppressive function and inhibits $CD8^+$ T cell proliferation and cytotoxic factor secretion.

Note: (A) CFSE dilution assay assessing $CD8^+$ T cell proliferation; scale bar = 50 μm. (B) Quantification of $CD8^+$ T cell proliferation index. (C) ELISA analysis of IFN-γ and Granzyme B secretion levels. (D) Flow cytometric analysis of CD69 expression in a Transwell co-culture system; scale bar = 50 μm. (E) Quantification of $CD69^+$ cell frequency. (F) ELISA quantification of IL-10 and TGF-β concentrations in Treg-conditioned supernatants. All experiments were repeated at least three times. ns, not significant; *$p < 0.05$; **$p < 0.01$; ***$p < 0.001$.

Figure S2. Foxp3 knockdown markedly attenuates the Treg-activating and neuroprotective effects of MOF@PI-IL2.

Note: (A) WB analysis of Foxp3 and p-STAT5 protein levels in Treg cells. (B) ELISA quantification of IL-10 and TGF-β secreted by Treg cells. (C) Whole-cell patch clamp recordings of action potential amplitude and firing frequency in DRG neurons. (D) Ca^{2+} imaging of Ca^{2+} peak amplitude, event frequency, and Ca^{2+} activity area; scale bar = 25 μm. (E) Immunofluorescence staining of GAP43 and NF200 expression; scale bar = 25 μm. All experiments were performed at least three times. ns, not significant; *$p < 0.05$; **$p < 0.01$; ***$p < 0.001$.

Figure S3. RNA-seq differential expression, immune infiltration profiling, and functional pathway enrichment analysis.

Note: (A) Volcano plot illustrating the distribution of DEGs between the Sham and DPN+NS groups based on RNA-seq analysis. (B) Clustered heatmap displaying the gene expression profiles of DEGs between the Sham and DPN+NS groups. (C) Immune cell proportion changes between the Sham and DPN+NS groups were analyzed by immune deconvolution using CIBERSORT. (D) Volcano plot showing the distribution of DEGs between the DPN+NS and DPN+MOF@PI-IL2 groups. (E) Heatmap presenting the gene expression patterns of DEGs between the DPN+NS and

DPN+MOF@PI-IL2 groups. (F) Immune infiltration analysis using CIBERSORT to determine immune cell composition differences between the DPN+NS and DPN+MOF@PI-IL2 groups. (G) GO enrichment analysis identifying the BP, CC, and MF associated with DEGs between the Sham and DPN+NS groups. (H) KEGG pathway enrichment analysis of DEGs between the Sham and DPN+NS groups. (I) GO enrichment analysis of BP, CC, and MF terms enriched among DEGs between the DPN+NS and DPN+MOF@PI-IL2 groups. (J) KEGG pathway enrichment analysis of DEGs between the DPN+NS and DPN+MOF@PI-IL2 groups.

Figure S4. RNA-seq deconvolution analysis of immune cell infiltration in neural tissues.

Note: (A) Heatmap visualization of immune cell infiltration levels in neural tissues from the Sham and DPN+NS groups, estimated by CIBERSORT, showing the relative abundance of distinct immune cell subsets. (B) Stacked bar plot illustrating the proportions of immune cell types in neural tissues of the Sham and DPN+NS groups based on CIBERSORT analysis. (C) Heatmap displaying the immune infiltration profiles of neural tissues from the DPN+NS and DPN+MOF@PI-IL2 groups as estimated by CIBERSORT. (D) Stacked bar plot showing the composition of immune cell populations in neural tissues of the DPN+NS and DPN+MOF@PI-IL2 groups according to CIBERSORT results.

Figure S5. Integrated metabolomic-RNA-seq analysis and WGCNA of neural tissues in DPN.

Note: (A) Volcano plot illustrating the distribution of differential metabolites between the Sham and DPN+NS groups. (B) Correlation heatmap showing metabolite-sample associations for differentially expressed metabolites between the Sham and DPN+NS groups. (C) Pearson correlation analysis assessing the association between SCFA levels and Treg-associated gene expression in the Sham and DPN+NS groups. (D) Pearson correlation analysis examining the relationship between Kyn levels and pro-inflammatory gene expression in the Sham and DPN+NS groups. (E) Pearson correlation analysis evaluating the correlation between Kyn levels and Treg-related gene expression in the Sham and DPN+NS groups. (F) Volcano plot displaying differential metabolite distribution between the DPN+NS and DPN+MOF@PI-IL2 groups. (G)

Correlation heatmap showing metabolite-sample associations for differentially expressed metabolites between the DPN+NS and DPN+MOF@PI-IL2 groups. (H) Pearson correlation analysis assessing the relationship between SCFA levels and Treg gene expression in the DPN+NS and DPN+MOF@PI-IL2 groups. (I) Pearson correlation analysis evaluating the association between Kyn levels and pro-inflammatory gene expression in the DPN+NS and DPN+MOF@PI-IL2 groups. (J) Pearson correlation analysis investigating the correlation between Kyn levels and Treg-associated gene expression in the DPN+NS and DPN+MOF@PI-IL2 groups. (K) Bar plot depicting gene significance scores across WGCNA modules in the Sham and DPN+NS groups. (L) Module-trait heatmap illustrating the correlations between WGCNA modules and immune infiltration characteristics in the Sham and DPN+NS groups. (M) Bar plot showing gene significance scores across WGCNA modules in the DPN+NS and DPN+MOF@PI-IL2 groups. (N) Module-trait heatmap displaying the correlations between WGCNA modules and immune infiltration features in the DPN+NS and DPN+MOF@PI-IL2 groups.

Figure S6. Standardized analysis of metabolomic data.

Note: (A) Distribution plots of the top 50 metabolites in the Sham vs. DPN+NS groups, before (left) and after (right) normalization. (B) Distribution plots of the top 50 metabolites in the DPN+NS vs. DPN+MOF@PI-IL2 groups, before (left) and after (right) normalization. (C) Soft-thresholding power selection plots displaying scale independence and mean connectivity to determine the optimal soft-threshold for WGCNA in the Sham and DPN+NS groups. (D) Gene dendrogram illustrating gene clustering and module identification in the Sham and DPN+NS groups. (E) Scatter plot showing the relationship between module membership and gene significance in the green module. The x-axis represents module membership, and the y-axis indicates gene significance. Pearson correlation coefficients and *p*-values are provided to denote statistical relevance. (F) Soft-thresholding power selection plots for WGCNA in the DPN+NS and DPN+MOF@PI-IL2 groups. (G) Gene dendrogram illustrating clustering structure and module classification for the DPN+NS and DPN+MOF@PI-IL2 groups. (H) Scatter plot depicting the correlation between

module membership and gene significance in the turquoise module, with axes defined as in (E) and accompanying statistical metrics.
